# Facilitators and barriers to online group work in higher education within health sciences – a scoping review

**DOI:** 10.1080/10872981.2024.2341508

**Published:** 2024-04-12

**Authors:** Live Edvardsen Tonheim, Marianne Molin, Asgeir Brevik, Malene Wøhlk Gundersen, Lisa Garnweidner-Holme

**Affiliations:** a Department of Nursing and Health Promotion, Faculty of Health Science, Oslo Metropolitan University, Oslo, Norway; b Division for Research, Development and University Library, Oslo Metropolitan University, Oslo, Norway

**Keywords:** Online group work, health education, online education, higher education, health science, community of inquiry framework, student group work, scoping review

## Abstract

**Introduction:**

In health education, group work is essential to prepare students for working in health care and medical teams. Following the widespread adoption of online teaching, group work increasingly takes place in online environments. Although successful group work can provide good learning outcomes, it is unclear what facilitates or hinders online group work in health science education, and to what extent this topic has been addressed. Thus, this scoping review aimed to identify the facilitators and barriers to online group work in higher health education, provide an overview of the scientific literature related to the topic, and identify knowledge gaps in the research.

**Methods:**

This scoping review was guided by the methodological framework described by Arksey and O’Malley, and reporting is in accordance with Preferred Reporting Items for Systematic Reviews and Meta-Analysis Extension for Scoping Review (PRISMA-ScR). Eight online databases were searched for scientific articles published between 2012 and 2022. At least two researchers independently screened records and full-text articles and charted data including article characteristics and key information related to the research question. Findings were categorized and summarized based on the Community of Inquiry Framework.

**Results:**

After screening 3671 records and 466 full-text articles, 39 articles met the inclusion criteria. The review revealed smaller group size, consistency in group composition and joint responsibility to be facilitators. Challenges with group communication, scheduling synchronous meetings and technical issues were identified as barriers. Our findings supported the importance of all three elements of the Community of Inquiry Framework: social, cognitive, and teaching presence.

**Conclusion:**

This review provides an overview of facilitators and barriers to online group work in health science education. However, there is a need for further investigation of these factors and studies addressing this topic from the teachers’ perspective.

## Introduction

Group work is a common teaching strategy in health education [1] and fundamental in several learning models frequently used. Working in groups may help students acquire team-building and collaboration skills that are essential when working in healthcare settings [1]. There is strong scientific support for the benefits of learning in groups [2–7]. However, group work is not always successful, and challenges can affect both learning outcomes and student satisfaction [2,6,8–11].

Following the wide adoption of online teaching, group work is increasingly taking place in online environments [12], and challenges in online group work in higher education, in general, have been thoroughly addressed in previous research [13]. Also, within health science education specifically, several studies have investigated different aspects related to online group work (e.g., communication [14], participation and distribution of workload [14,15], technical challenges [14], the teacher’s role [15–17] and how groups are created [15]). However, there is to our knowledge no available overview of the literature on online group work in health science education.

Online group work may cover various forms of group work and online environments. For this scoping review, we chose to define online group work as ‘not in-person’, meaning group taking place solely online. Furthermore, we defined group work as independent student work without a monitor or teacher present.

### The community of inquiry framework

Originally developed focusing on asynchronous online learning [18], the Community of Inquiry (CoI) framework has since been applied to various online learning modes, including synchronous and blended [19]. It has been widely used to describe, implement and evaluate collaborative online learning [18–22] in research covering students’ learning experiences [22], attitudes [23] and perceived and actual learning [24]. Founded on Dewey’s educational philosophy and social constructivism, the framework is described as a collaborative constructivist model of learning and education (20, chpt. 2).

**Figure 1.**
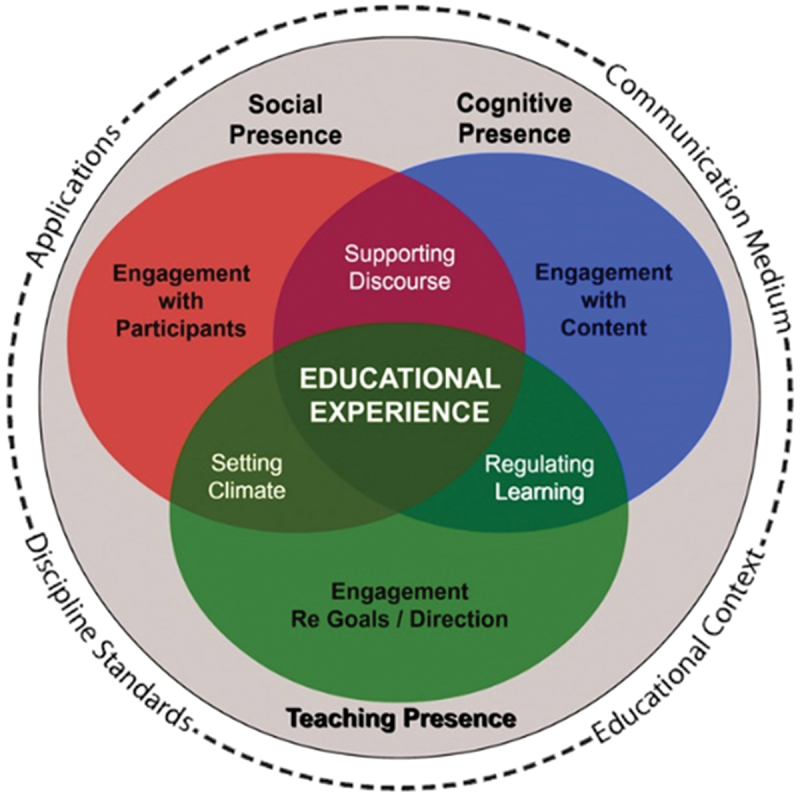
The community of inquiry framework. Image used with permission from the community of inquiry website and licensed under the CC-BY-SA International 4.0 license (https://creativecommons.Org/licenses/by-sa/4.0/). The original image is located at https://www.thecommunityofinquiry.org/framework.

The core premise of CoI is that learning is best promoted in a community of learners that engage in critical discourse, a ‘community of inquiry’, which is reinforced by three interacting elements: Social presence, cognitive presence, and teaching presence (Figure 1). These three elements are further described and operationalized through the categories used in the Community of Inquiry Survey (CoI Survey) [25], which has been validated through multiple studies in different contexts [19]. Social presence, engagement with participants, exists in a trusting environment that encourages critical discussion and may be identified in forms of the categories affective communication (the social emotional environment), open communication (in a climate of trust), or cohesive communication (group cohesion and sense of belonging), all described in the CoI Survey (20, chpt. 4, 25). Cognitive presence, engagement with content, is operationalized through the model of practical inquiry. The practical inquiry model describes the process of constructing meaning through reflection and discourse in four phases used as categories in the CoI Survey: triggering event (initiation and presenting the problem), exploration (searching for information and understanding the problem), integration (gaining insight and constructing meaning), and resolution (reducing the complexity or reaching a solution of the problem) (18, 20, chpt. 5). Teaching presence, engagement regarding goals and direction, is divided into three categories and in the CoI Survey: design and organization, facilitating discourse, and direct instructions (20, chpt. 3, 25). Teaching presence can be described as the educator’s role of planning and delivering education but may also be found in what learners do to create an environment of social and cognitive presence (20, chpt. 6).

### Study objectives

Understanding what facilitates or hinders online group work is key to creating well-functioning groups and group assignments as well as efficient methods for monitoring and facilitating. All of which may contribute to successful group work, leading to good learning outcomes and student satisfaction. To our knowledge, no literature has comprehensively explored the potential facilitators and barriers to online group work in health science education. This scoping review aims to address this gap and identify potential facilitators and barriers to online group work in higher education within health sciences. Our findings will be presented and discussed in the light of the CoI framework. Due to the broad nature of the aim, we considered the scoping review the best-suited approach.

To guide the scoping review, we developed the following research questions:

1. What are the facilitators and barriers to online group work in higher education within health sciences?
2. What kind of scientific publications related to facilitators and barriers to online group work in higher education within health sciences are available, and what are the knowledge gaps in the scientific publications related to facilitators and barriers to online group work in higher education within health sciences?

## Methods

We conducted this scoping review following the steps proposed by Arksey and O’Malley [26] and Levac et al. [27] and used The Preferred Reporting Items for Systematic Reviews and Meta-Analysis Extension for Scoping Review (PRISMA-ScR) for reporting [28]. The protocol is available from the Open Science Framework https://osf.io/da57b/

### Information sources and search

The search strategy was developed in collaboration with the experienced health science librarian on the research team and adapted across each of the eight included databases (MEDLINE via Ovid, EMBASE via Ovid, PsycINFO via Ovid, ERIC via EBSCOHost, Education source via EBSCOHost, CINAHL via EBSCOHost, Scopus and Epistemonikos).

Search strings covering four dimensions (online learning environment, group work, higher education and health sciences) were combined using Boolean operators. The search was limited to articles written in languages all members of the research team could read (Danish, English, Norwegian, and Swedish), and to studies published in the last 10 years, due to the rapid technological developments. The full search strategy for the search is in Appendix 1. The search was performed on 2 September 2022 in all eight databases. The reference lists of the included articles were screened for additional studies, and two selected journals (Nurse Education Today and BMC Medical Education) were hand-searched.

### Selection of studies

After duplicates were removed, the search results were uploaded to Rayyan [29]. To increase consistency among reviewers, at least three members of the research team screened the same 50 titles and abstracts individually before discussing uncertainties surrounding inclusion and exclusion criteria. The remaining titles and abstracts were screened individually by two researchers, with one researcher screening all titles and abstracts. Any disagreements were resolved by a third researcher. All researchers met regularly throughout the screening process to discuss and refine any unclear criteria. The same approach was applied in screening of included full-text versions of the articles.

### Eligibility criteria

The eligibility criteria were based on the research questions and insights from preliminary searches (January 2022 and April 2022). Following the recommendations of Levac et al. [27], the criteria were developed through an iterative process with repeated discussions throughout the screening process, and aimed to locate scientific articles that addressed the population, concept, and context as outlined in Table 1. The concept of group work was defined as independent student group work without monitor or teacher present, and the context was defined as solely online, meaning ‘not in-person’ group work. All types of study designs were considered.

**Table 1.** Eligibility criteria based on population, concept, context, and main aim.

	Inclusion	Exclusion
Population (Students in higher education within health sciences)	Students (any age) in higher education within health sciences Students in programs/courses in health care and health sciences (including education of authorized health personnel)	Students in non-credited higher education courses/programs – (i.e., continuing education/short courses at the workplace) Students in programs/courses not within health sciences Interns or residents (graduated from medical school) are the only participants PhD-fellows/candidates Veterinary students
Concept (group work)	Studies specifically addressing independent group work as a voluntary or obligatory learning activity in credited higher education within health sciences. Including but not limited to collaboration, cooperation, discussions and tasks and work assignments Studies addressing learning models in which independent group work is a fundamental part	Studies not specifically addressing group work Studies addressing learning models in which group work is not essential Small group teaching (instructor/tutor/facilitator/teacher continuously present as part of the group)
Context (online)	All variations of students working together in digital learning contexts/environments including but not limited to synchronous, asynchronous, blended or hybrid solutions. Group work takes place solely online meaning ‘not in-person’	Fully physical learning environment Student’s group work taking place in-person (even if preparations or part of the course are online)
Facilitators or barriers to online group work	Reporting findings on student or faculty’s experience, opinions of potential facilitators or barriers to online group work Reporting effect or associations between potential facilitators or barriers and online group work	Not addressing or reporting any potential facilitators or barriers to online group work Group work is regarded as a facilitator or barrier to learning in a course, but has not been investigated further

### Data charting process and data items

We developed a data charting form including article characteristics (e.g. first author, year of publication, country, study aim, design, methodology, participants, and main study outcomes) and key information related to our research questions (e.g. online mode, group characteristics, type of group work, and findings related to facilitators and barriers in online group work) (Appendix 2). Data specifically relevant to the main aim was charted based on the CoI Framework. Two reviewers independently charted the data. After charting data from 5 articles, the two reviewers discussed and resolved any unclarities regarding the criteria to enhance consistency. Any disagreements were solved by a third reviewer. Charting the data was treated as an iterative process, allowing the data charting form to be updated if it became apparent that additional data could be usefully charted.

### Synthesis of results

Data were categorized and synthesised descriptively using the CoI Framework and Survey as a guideline for the categories [20,25]. To include data that did not fit within the three elements (presences) a category ‘Facilitators and barriers outside CoI’, referring to the contextual factors surrounding the inner circles of the framework (Figure 1) was added. We chose the CoI Framework, in consultation with stakeholders, because it captures a wide range of aspects relevant to the online learning context and to group work [19,24]. As the framework encompasses overarching elements and more detailed categories, it was considered well suited to provide structure to the data we anticipated to be potentially widespread due to the broad scoping review method. Also, we considered the distinct and yet interrelated presences would give us a perspective from which to look for connections and commonalities. Moreover, as we wanted to include all study designs and methods, we considered it an advantage that the framework has been used in education research using different methodologies within qualitative [22], quantitative [19,24,30] and mixed methods [22] research.

Following recommendations by Colquhoun et al. [31], we consulted stakeholders to gain a broader understanding of our findings and enhance the study’s validity.

## Results

### Sources of evidence

**Figure 2.**
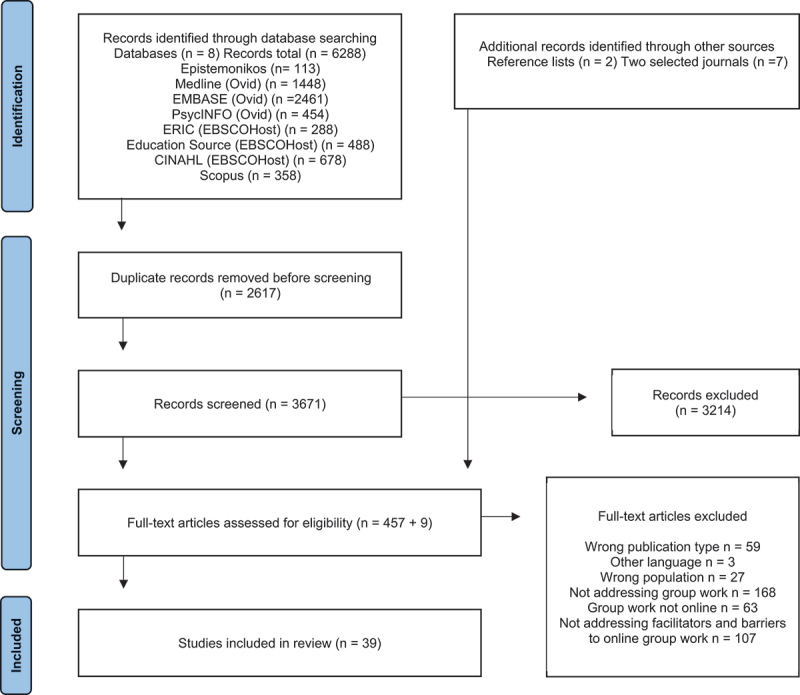
PRISMA flow chart of the study selection.

Following database searching, screening, and data charting, a total of 39 articles met the inclusion criteria (Figure 2). The study characteristics for all included studies are presented in Table 2. The articles were published from 2012 to 2022. By country, the highest represented was the United States (*n* = 15), followed by Australia (*n* = 4), Spain (*n* = 3), and collaborative studies between the UK and Somaliland (*n* = 3). Most studies used both quantitative and qualitative methods (*n* = 24), and articles using qualitative methods exclusively (*n* = 10) were twice those using quantitative methods only (*n* = 5). Quantitative data were mostly collected through questionnaires (*n* = 23), or recorded grades or scores on tests or assignments (*n* = 5), and qualitative data were collected through open-ended questionnaire items (*n* = 15), focus group interviews (*n* = 9), student-written documents or messages (*n* = 6) or individual interviews (*n* = 3). Other types of data included grades or test scores (*n* = 5). The varying modes of online group work assessed included asynchronous (*n* = 6), synchronous (*n* = 13), asynchronous and synchronous combined (*n* = 11), and different forms of blended or hybrid solutions (*n* = 4). The duration of the online group work spanned one session to one semester, and potential facilitators and barriers to online group work were addressed as a main aim in six articles. Most articles concerned nursing students (*n* = 18), medical students (*n* = 14), or pharmacy students (*n* = 7). Several articles reported to include students from more than one health program (*n* = 7).

**Table 2.** Study characteristics of 39 included articles.

First author (year) Country	Purpose	Design and data collection methods	Health professional study field	Online mode	Type of assignment	Duration of group work	Group size
Abdelhakim, H. E [32]. (2022) UK	*ʽTo explore student’s perspectives towards IPE education and to use that information to improve the design of these sessions in the future.ʼ*	Quantitative and qualitative; Surveys (Likert scale and open-ended)	Pharmacy (MPharm), Medicine and Surgery (MBBS)	Synchronous	Case studies, discussion (second case in breakout rooms)	3-hour session	≤8
Adwan, J [33]. (2016) US	*ʽTo investigate the effectiveness of using a dynamic Google Forms peer evaluation form in online group activities in improving group work outcomesʼ*	Descriptive comparative design; Surveys (Likert scale and open-ended)	Nursing students – Health informatics course	Synchronous and asynchronous	Produce online Voice Thread presentations	1 semester	5
Ambrose, M [34]. (2017)Australia and Indonesia	*ʽTo explore the experiences of medical students in Australia and Indonesia who participated in a reciprocal intercultural participatory peer e-learning activity (RIPPLE) in global health.ʼ*	Surveys (Pre- and post; Likert scale and open-ended)	Medical	By choice	Produce a 500-word structured literature review in abstract format	2 weeks	≤8
Breen, H [35]. (2015)US	*ʽUnderstanding the usefulness of Online Collaborative Learning Theory (Harasim) for the assessment of collaborative discussions in nursing education.ʼ*	Qualitative Constant comparative Transcript analysis	Nursing	Asynchronous (discussion forums)	Development of nursing action plan to respond *to the needs of agreed-upon members of the virtual community*	1 week following 1 week in whole class collaboration	4–5
Bristol, T. J [36]. (2012)US	*ʽTo explore the effect of size and strategy on asynchronous discussions in a small baccalaureate nursing program.ʼ*	Descriptive correlational and quasi-experimental methodology; Surveys (Likert scale and open-ended)	Nursing, Second-semester adult medical-surgical course and obstetrics/pediatrics course	Asynchronous	Asynchronous discussion homework assignments	16-week semester	11 and 12 vs. 23
Bybee, S [37]. (2022) US	*ʽTo [1] address health science students’ attitudes toward group work and peer assessment [2], apply evidence-based recommendations and student suggestions to the adaptation of a PAR, and [3] determine the rubric’s effectiveness for educators and acceptability among students.ʼ*	Evaluation; pre- and post-surveys	Nursing, Pharmacy, Dental and Medical students 1) Palliative care course 2) Ambulatory care course (Zoom- ambulatory)	Prework asynchronous, synchronous	Ambulatory: Group session in 4-hour workshop- in person or virtually Palliative care: Group work incorporated throughout the semester	Ambulatory: Group session in 4-hour works Palliative care: Throughout the semester	5–7 (Ambulatory groups)
Darici, D [38]. (2021) Germany	*ʽTo evaluate the implementation of a histology course previously taught in a classroom setting into an online-only format based on video conference software.ʼ*	Evaluation; pre- and post-surveys (Likert scale and open-ended)	Medical, digital Histology course	Synchronous	Work-sessions in smaller groups (breakout rooms) – solving specific problems.	10–25 minutes per group session, 2–3 group sessions per course session throughout 1 semester (19 course days)	5–10
Ferguson, C [39]. (2016) Australia	*ʽTo explore first year Bachelor of Nursing student nurses’ experiences with social media in supporting student transition and engagement into higher education.ʼ*	Qualitative; Focus group interviews.	Nursing	Asynchronous and synchronous	NA	NA	NA
Floren, L. C [40]. (2020) US	*ʽTo develop and evaluate a mobile learning module to support knowledge construction between medical and pharmacy students through structured dialogue prompts.ʼ*	Quantitative and qualitative feedback on CBL; Scoring student work	Medical and pharmacy	Asynchronous	Virtual interactive case in three phases,	two-weeks	2
Ganotice, F. A [41]. (2022) China	*ʽTo distinguish the characteristics of high- and low-performing teams, and examine whether self-determined motivation could predict group membership in high- and low-performing teams.ʼ*	Quantitative; Survey and test scores	Chinese Medicine, Medicine, Nursing, Pharmacy, Undergraduate Social Work and Master’s Social Work, Interprofessional educational module	Asynchronous and synchronous	Work as a team to solve a complex problem (Case-based learning, Care plan, independent team meeting, breakout meeting, peer-evaluation)	10 days	5–8
Garratt-Reed, D [42]. (2016) Australia	*ʽTo compare student grades and satisfaction, as well as retention rates, in online and face-to-face versions of an introductory psychology unit.ʼ*	Quasi-experimental methods; Marks; Student feedback (Likert scale and open-ended)	Psychology	Asynchronous	Group Presentation Assignment	Submission before the end of week 12, but not stated if the assignment ran from the start of the semester.	Not stated, student comments suggest one group was approximately 3 students
George, T. P [43]. (2019) US	*ʽTo determine student perceptions about collaborative learning activities between prelicensure BSN and MSN students.ʼ*	Survey (Likert scale and open-ended)	Nursing	Hybrid or blended asynchronous – BSN students on campus, MSN students online	Create low-literacy pamphlets for community sites based on community assessment. Use pamphlets in educational sessions at community sites	1 semester	Not stated
George, T. P [44]. (2021) US	*ʽTo implement a virtual synchronous debate in an online graduate nursing course and to determine whether students’ ability to communicate professionally about ethical issues in health care improved after participating in the learning activity.ʼ*	Survey (Likert scale and open-ended)	Nursing, course in advance practice nursing	Synchronous	Online debate	1 semester: 1 debate session (active participation) 2 debate sessions (attendance only)	3
Govindarajan, S [45]. (2022) India	*ʽTo implement and evaluate online TBL among first year MBBS students in Biochemistry.ʼ*	Implementation and evaluation of TBL; Feedback (Likert scale and open-ended)	Medical, Bachelor of Surgery (MBBS) Biochemistry course	Synchronous	Online TBL session	3 sessions, 2 hours	10
Hastuti, A. A. M. B [46]. (2022) Indonesia and foreign countries (Vietnam, Philippines, Malaysia, Cambodia, Taiwan, Japan, Nepal, Pakistan, Iraq, Uganda, Saint Kitts and Nevis)	*ʽTo determine the feasibility of using the virtual world platform of SL to deliver an IPE experience focused on palliative care topics.ʼ*	Mixed methods; Questionnaire (Likert scale and open-ended)	Medical, Nursing, Nutrition, Physical Therapy and Social work students, Online summer course on traditional medicine	Synchronous and asynchronous	Discussion sections in synchronous activities and asynchronous project assignments	10 working days, 9 hours per day	Not stated
Haugland, M. J [47]. (2022) Norway	*ʽTo describe, explore and discuss how the students collaborated in small groups in an online course to learn.ʼ*	Case study; Focus group and individual interviews	Philosophy of science, ethics, and research methods, Master’s-level health programs	Synchronous and asynchronous	Problem-based assignments	Not stated	3–5
Hovlid, E [48]. (2022) Norway	*ʽTo explore students’ experiences and perceptions of learning Quality improvement (QI) competence after participating in the virtual learning module, and to identify factors that promote or inhibit virtual learning of QI competence*.ʼ	Exploratory case study design; Focus group interviews.	Healthcare and social services postgraduate course, Health professionals	Synchronous	Case and presentation	1 day	4–6
Keynejad, R [49]. (2013) UK and Somaliland	*ʽTo share knowledge and experiences between medical students in Somaliland and the United Kingdom, for psychiatry education and cross-cultural exchange … . assess the usefulness and feasibility of online, instant messenger, peer-to-peer exchange for education in psychiatry between cultures.ʼ*	Questionnaires and surveys (Likert scale and open-ended)	Medical, Psychiatry	Synchronous	Discussions of suggested themes (psychiatry topics)	Eight 1-hour meetings	2
Keynejad, R [50]. (2016) UK and Somaliland	*ʽFirst to examine attitudes to psychiatry pre- and post-participation in peer-to-peer global mental health e-learning; second, to examine stigmatized attitudes in medical students pre- and post-participation; and third, to assess students’ subjective reports of learning arising from this partnership. This study had two further methodological aims: to qualitatively assess the practical applicability of this e-learning model and to explore improvements to address implementation problems.ʼ*	Mixed methods; Survey (Likert scale and open-ended)	Medical	Synchronous	Discussions of suggested themes (psychiatry topics)	Ten 1-hour meetings every second week	2
Kor, P. P. K [51]. (2022) China	*ʽTo explore undergraduate nursing students’ self-regulated learning experiences, satisfaction, and attitudes toward older persons in a gerontological nursing course using online self-regulated enquiry-based learning (EBL) during the COVID-19 pandemic.ʼ*	Sequential explanatory design; Surveys (Pre- and post, Likert scale); Focus groups	Nursing, Gerontological nursing course	Synchronous (and asynchronous? Not clearly stated)	Using video-based scenarios: Explaining the problem, develop mind map and discuss it with peers, analyze the problem, find solution, present evidence.	3 months	10–12
Kwon, K [52]. (2014) US	*ʽExplored what social interactions students exhibited during collaborative learning, and analyzed how the social interactions evolved in a computer-supported collaborative learning (CSCL)* *environment.ʼ*	Ex post facto design; Descriptive analysis; Cluster analysis	Health science	Asynchronous	Online discussions and Wiki. To develop a clinical ethics case scenario and act out the roles of the stakeholders.	16 weeks (1 semester)	5–6
Liu, Y-H [53]. (2018) US	*ʽTo answer the following questions*: *1. Are there differences between groups in terms of individual work experience, online learning experience, and group process in early collaboration?* *2. How were the individuals’ work experience, online learning experience, and the group process in the early collaboration phase associated with student* *perceptions of the overall group collaboration.ʼ*	Ex post facto design; Surveys (Likert scale)	Prehealth professional programs, Course in clinical ethics required in	Not stated	Co-writing a Clinical ethics case	Week 4–16 in a 1 semester, 16 weeks course	3–6
Mayordomo, R. M [54]. (2015) Spain	*ʽTo (i) identify the processes of collaborative knowledge construction and the processes of organization and coordination of work in a small group collaborative virtual task which has a written product; (ii) compare both types of processes among groups with low and high levels of performance in the task; and (iii) explore possible relationships between the types of organization and coordination of work used by the groups and the levels of collaborative knowledge construction they reach in the development of the task.ʼ*	Single case exploratory study; Students’ documents and written communication	Instructional psychology course	Not stated, but asynchronous is implied	Small group virtual task The final product of the task was a global report on the work done and the results obtained	5 weeks	4
Nicklen, P [55]. (2016) Australia	*ʽTo compare the effect of remoteonline CBL (ROCBL) with traditional facetoface methods on learning outcomes of undergraduate physiotherapy students. The secondary aims were to explore student satisfaction and response, and perceived depth of learning with ROCBL.ʼ*	RCT; Survey; Focus group interviews	Physiotherapy	Synchronous via web-conferencing	CBL sessions	2 CBL sessions (approximately 90 min per session) and outcome assessment, 1 week	4–6 *(only stated in control)*
Onrubia, J [56]. (2015) Spain	To ʽ*present a teaching innovation experience that sought to promote individual and group regulated learning in university students who work in a computer- mediated collaborative environment. ʼ*	Teaching innovation intervention; Questionnaire (Likert scale and open-ended)	Psychology, Educational psychology course	Blended	Topic based case studies and creation of conceptual maps	12 weeks *(three topic areas, each lasting about four weeks)*	4–6
Palmer, R. H [57]. (2022) US	*ʽTo evaluate the impact of synchronous hybrid instruction on pharmacy students’ engagement in a pharmacotherapy course.ʼ*	Mixed methods; Survey (Likert scale and open-ended)	Pharmacy, Pharmacotherapy course at College of Pharmacy	Synchronous hybrid	Develop patient care plans	1 semester	4–6
Peterson, A. T [58]. (2016) US	To ʽ*examine the independent and interactive effects of four CSCL strategies: social interdependence, summarizing, scripts, and synchronicity.ʼ*	Experimental-control design; Questionnaires (Likert scale)	Nursing, online graduate nursing course	Synchronous, asynchronous	Case studies	16-week semester	3–5
Prosser, M [59]. (2021) UK and Somaliland	*ʽTo explore the experiences of students participating in the Aqoon programme, including their experiences of reflective practice.ʼ*	Qualitative; Students’ writing and focus group interviews	Medical (medical school)	Synchronous and asynchronous	Case-based discussions	1-hour sessions weekly, 7 weeks	3 (2 from Somaliland, 1 from UK)
Raymond, A [60]. (2016) Australia	*ʽTo examine students’ opinions of the experience of peer group online learning.ʼ*	Qualitative; Questionnaire (Open-ended)	Nursing, Pharmacology course	Not specified	Activities in group online forum sessions and assessment tasks	Not stated	6–8
Raynault, A [61]. (2021) Canada	*ʽTo 1) demonstrate how interprofessional teams of students mobilize framework competencies and care approaches during online and face-to-face collaborative learning activities; and 2) analyze how students collaborate during a hybrid IPE course using a patient partnership approach.ʼ*	Qualitative; Students’ work and questionnaire (Open-ended)	Audiology [1], occupational therapy [7], kinesiology [1], medicine [11], dental medicine [1], nursing [14], nutrition [3], optometry [3], speech therapy [2], pharmacy [9], physiotherapy [1] and social work [6] programs.	Synchronous, asynchronous and face-to-face workshop	Co-create two activities (a therapeutic education and an interprofessional vignette) in the online collaborative journal.	Not stated	5
Rogo, E. J [62]. (2014) US	*ʽTo explore student experiences in a graduate dental hygiene program contributing or impeding the development and sustainability of online learning communities.ʼ*	Qualitative Case study; Individual interviews	Dental hygiene	Asynchronous, in addition to 2 campus visit (1 week each)	Group activities and projects, or peer review activities.	Not stated (group activities and projects, or peer review activities throughout the 3-year graduate program)	Not stated
Ropero-Padilla, C [63]. (2021) Spain	*ʽTo explore nursing students’ experiences and perceptions of the use of game elements in two full-nursing subjects using a blended-learning teaching strategy.ʼ*	Qualitative; Focus group interviews	Nursing students, Adult and Elderly Health Programmes and Chronic Processes	Blended, online and practice classes	Team-based activities and Team-based game sessions	45 hours distributed over 23 sessions	5–6
Sand, J [64]. (2021) US	*ʽTo describe an implemented online interprofessional course that meets the needs of undergraduate students from a variety of clinical and nonclinical health programs.ʼ*	Course evaluation; Questionnaire (Likert sand open-ended)	Health information management (HIM), interprofessional capstone course, including environmental and occupational health, HIM, health studies, nursing, public health, radiologic sciences, and respiratory care.	Not stated	*ʽTeamwork throughout the courseʼ: ʽdiscussions, and meetings, agreeing on a team norms document, and creating patient education materials.ʼ*	Throughout the course	Not stated
Shimizu, I [65]. (2022) Japan	*ʽTo compare the degree of social interdependence in OCL with face-to-face environments and clarify aspects that affect social interdependence in OCL.ʼ*	Mixed methods RCT (crossover); Questionnaire (Likert scale), and Focus group interviews	Medical	Synchronous	Group discussions as a team as part of TBL. Tasks regarding problem solving of medical cases	TBL process 6 weeks online, 6 weeks face-to-face	7–9
Siah, C.-J. R [66]. (2022) Singapore	*ʽTo examine the impact of online-tutorials in place of face-to-face tutorials on knowledge level and understand the perspectives of learners who experience online-tutorials.ʼ*	Mixed methods experiential design; Assignment and exam results, and Individual interviews	Nursing, Psychology for Nurses module	Synchronous	Group written assignment, discussions	2-hour sessions from week 1–13	Smaller groups of 5 implied in results
Trobec, I [67]. (2015) Slovenia	*ʽA pedagogical experiment conducted to identify the readiness and responsiveness of current organisation of nursing higher education in Slovenia through It compared the successfulness of active learning methods online (experimental group) and in the traditional classroom (control group) and their impact on the ethical competences of nursing students. ʼ*	Non-randomized interventional study; Questionnaire (Likert scale and open-ended)	Nursing, Philosophy and Professional Ethics in Nursing course	Synchronous and asynchronous	Discussions in tutorials	Not stated tutorials were part of the course (The study was performed in the academic year 2010/2011)	5–6
Vogt, M. A [68]. (2016) US	*ʽTo evaluate various technology enhanced methods on the learning acquisition and satisfaction of graduate nursing students in an advanced pharmacology class using an unfolding case study.ʼ*	Mixed methods; Group grades and survey (Likert scale and open-ended)	Nursing, Advanced pharmacology course	Synchronous or asynchronous	Evolving case	3 weeks	Five groups of 8 and one group of 6
Wolcott, M. D [69]. (2022) US	*ʽTo describe the implementation and impact of an introductory session on psychological safety for incoming first-year Doctor of Dental Surgery (DDS) learners.ʼ*	Intervention, Mixed Methods; Surveys (Different scales and open-ended), Students’ work, and Focus group interviews	Dental	Synchronous	Small group conversations	2 hours session	8–10
Yamashita, T [70]. (2021) Japan	*ʽTo evaluate the utility of online group work in interprofessional education.ʼ*	Quantitative study; Test scores	Medical and nursing	Synchronous	Discussion activities ‘NASA Exercise: Survival on the Moon’	1 session	4–5

### Synthesis of results

We categorized and summarized our data based on the three presences and their categories according to the template in the CoI Framework (20, chpt 3) based on the CoI Survey [25]: Social presence, cognitive presence, and Teaching presence adding a category for the facilitators and barriers outside the framework (Table 3).

**Table 3.** Facilitators and barriers to online group work in health science education categorized according to the community of inquiry framework.

		Facilitators	Barriers
Social presence	Affective expression	Getting to know the other group members [66] Establishing and improving interpersonal relationships [43,67] Working in the same group over time [47,48,63]	Lack of personal interaction in group work [64]
	Open communication	Feeling included and safe in the group [48] Trusting the others in the group [48] Reciprocity [54] Establishing quality communication early in the group process [52,53]	
	Group cohesion	Joint accountability, responsibility and shared commitment [45,47,48,52,54,63]. Equal distribution of workload [37,62] Establishing group norms [53] Intensive interactions early in the collaboration [52] Encouraging interaction and inclusion of all members of the group [67] Continuous socio-emotional interaction [48,52] Endorsing and valuing teamwork [41]	Lack of shared commitment [54] Working on tasks regardless of other group members [47] Unequal distribution of workload [42,44,48,62,64,68] Unequal distribution of responsibility [62] Limited interaction with the other group members [52].
Cognitive presence	Triggering events	Guidance prompts or questions [40,59] Micromovies/videos [51,69]	
	Exploration and integration	Joint responsibility, joint discussion and mutual revision [47,54] Assignments that promote sharing experiences and exploring one’s own and others’ opinions or perspectives could be seen as potential facilitators of exploration [68].	Dividing tasks between group members working individually [47,54]
Teaching presence	Design and organization	Smaller groups [36,62,64] 3–4 students per group [38,47,59] Choosing their team [63] Being in the same group over time [47,48] Groups with diversity in health professional fields [46,70] Problem-, or case-based assignments [47,48] Access to relevant learning resources before and throughout group work was found to be beneficial [32,51] Activities or requirements that supported group organization and regulation [56,58] Peer-to-peer feedback [33] or expectations of peer assessment [37],	Lack of time management for groups could be a potential barrier [45].
	Facilitating discourse	Discussion prompts seemed to enhance achievement [40,59].	Unclear instructions and explanations were suggested as a potential barrier in several studies [32,44,46].
Outside CoI		Agreeing on digital tools or social media between students to use for communication and collaboration [61] Using well-known digital tools or social media [39,49,50,60,61]	Technical challenges [50,57,60] Internet connection [34,49,59,63] Lag time [55] Insufficient instruction or competence in using technology as a potential barrier [60,65,68] Scheduling group meetings as a barrier [34,43,44,50,61,62,64,68] Find time to work individually on group assignments [35]

### Social presence

Social presence is described through identification of different types of communication. Our findings suggest that lack of communication in general [34,46,64] and challenges adapting to online communication [65,66] may be barriers to online group work. In the study by Ambrose et al. [34], qualitative findings revealed students’ frustration about establishing communication. Language was mentioned as a potential barrier in two studies, where some group members had to speak a second language [46,49].

#### Affective expression

Getting to know the other group members [66], establishing and improving interpersonal relationships [43,67], and working in the same group over time [47,48,63] were identified as potential facilitators for online group work influencing the learning outcomes [67] and academic performance [63]. Students expressed that being in the same group was important to make the working process more predictable [47] and create stability making it easier getting to know the others, which promoted the learning capacity [48]. Using a quantitative cluster analysis, Kwon et al. [52] found continuous interaction of encouragement between group members to be beneficial. On the opposite, results from a mixed methods study suggest that a lack of personal interaction in group work is a potential barrier [64]. Also, the quality of social interaction positively influences perceived learning [53].

#### Open communication

Students expressed that feeling included and safe in the group [48] was important to participate. Also, trusting the others in the group [48] and reciprocity [54] were important to foster communication, and establishing quality communication early in the group process [52,53] was found to facilitate group work. One of the barriers mentioned by students in several studies was the lack of non-verbal communication [48,65,67]. Closely related was not turning on the cameras [65], which further impaired non-verbal communication. Poor communication as well as completing tasks at the last minute could lead to broken trust [52].

#### Group cohesion

Joint accountability, responsibility, and shared commitment among the group members were identified as potential facilitators related to the sense of cohesiveness and belonging to a community [45,47,48,52,54,63]. Likewise, working on a common project [44] and equal distribution of workload [37,62] was reported as beneficial. Findings from a quantitative study suggested that establishing group norms [53] and having intensive interactions early in the collaboration [52] could facilitate the group process. Encouraging interaction and inclusion of all members of the group [67] and continuous socio-emotional interaction [48,52] were seen as important for successful group work. Also, endorsing, and valuing teamwork was linked to high performance in teams [41]. However, lack of shared commitment [54] in working on tasks regardless of other group members [47], as well as unequal distribution of workload [42,44,48,62,64,68] and responsibility [62] could be potential barriers. Limited interaction with the other group members in general and socio-emotional interaction were linked to ill-advised collaboration patterns [52].

### Cognitive presence

#### Triggering events

Guidance prompts or questions related to the assignment topic [40,59] were found to be potential facilitators acting as triggering events. Likewise, micro movies were appreciated [51] and videos were wanted to raise interest [69].

#### Exploration and integration

In two qualitative studies, organization of group work processes were reported to influence group discussions and collaborative learning processes. While ways of organization that required joint responsibility, joint discussion and mutual revision were seen as potential facilitators of exploration and integration processes in the groups [47,54], dividing tasks between group members working individually did not promote these processes [47,54]. Furthermore, assignments that promote sharing experiences and exploring one’s own and others’ opinions or perspectives could be seen as potential facilitators of exploration [68].

### Teaching presence

#### Design and organization

Related to assigning groups, students preferred smaller groups [36,62,64] of 3–4 students per group [38,47,59], choosing their team [63] and being in the same group over time [47,48]. Also, findings suggest that composing groups with diversity in health professional fields [46,70] may be a facilitator.

Problem-, or case-based assignments [47,48], and having access to relevant learning resources before and throughout group work were found to be beneficial [32,51]. Including activities or requirements that supported group organization and regulation [56,58] seemed to enhance achievement. However, lack of time management for groups could be a potential barrier [45]. Peer-to-peer feedback [33] or expectations of peer assessment [37], as well as making rubrics for group assignments was found to facilitate the group work. However, studies also reported inconclusive results related to whether peer evaluation could be a potential facilitator or barrier [33,45].

#### Facilitating discourse

Providing discussion prompts seemed to enhance achievement [40,59]. Unclear instructions and explanations were suggested as a potential barrier in several studies [32,44,46]

## Facilitators and barriers outside CoI

Technical challenges [50,57,60] including internet connection [34,49,59,63] and lag time [55] were frequently mentioned by students as potential barriers to online group work. Students also reported insufficient instructions or competence in using technology as potential barriers [60,65,68]. In several studies, students experienced scheduling group meetings as a barrier [34,43,44,50,61,62,64,68], and even to find time to work individually on group assignments [35]. Using well-known digital tools or social media to communicate and collaborate [39,49,50,60,61] were potential facilitators while failing to agree on means of communication was a potential barrier.

## Discussion

### Main findings

This scoping review identified facilitators and barriers to online group work in health education. Our analysis revealed smaller group size, consistency in group composition and joint responsibility as important facilitators for online group work in higher education within health sciences. Challenges with group communication, scheduling synchronous meetings and technical issues were identified as barriers. In the following, we discuss the most prominent facilitators and barriers from our analysis and previous research.

#### Through the lens of the CoI framework

As described in the introduction, the three presences in the CoI framework (social, cognitive, and teaching presence) are interdependent (20, chpt. 4). Due to this close relationship between the presences, we found some facilitators and barriers difficult to place within a single presence. Factors that potentially could impact or express more than one presence were therefore reported in all relevant presences. Although technical challenges and the use of social media or digital tools may influence all three presences, we interpreted these factors to be part of the educational context and communication media as outlined in Figure 1.

Our scoping review revealed facilitators and barriers that supported the importance of all three presences in the CoI Framework. However, most factors indicated social presence as described by Garrison as *‘… the ability of participants to identify with a group, communicate openly in a trusting environment, and develop personal and affective relationships progressively by way of projecting their individual personalities’* [71]. Exemplifying how social presence promotes cognitive presence (20, chpt. 4), we found that students needed to know each other, establish personal relationships, and feel safe before participating in group work [47,48,63,67]. Further, we found establishing relationships and communication to be particularly important in the early stages of group work. This is in line with research that shows social presence to be most prominent at the beginning while the balance shifts towards more cognitive presence as the group work progresses [72]. It also supports the importance of consistency in group composition, as this will allow social presence to be established in the group.

Most of our findings within teaching presence fell into the category ‘Design and organization’. This may be because we chose to define group work as students working independently in the group without a tutor or teacher always present. Thus, we may have excluded studies more likely to have investigated aspects of facilitating and directing the group work. Our findings related to cognitive presence were mostly concerned with organization, either regulated by the group alone or through an assignment, and learning activities that functioned as triggering events or facilitated exploration (early phases of practical inquiry). This corresponds to previously revealed challenges with reaching the more advanced phases of practical inquiry (integration and reconciliation) (20, chpt. 5). Also underlining the presences' interdependence, joint responsibility was found to facilitate both exploration in cognitive presence [47,54] and group cohesion in social presence [45,47,48,52,54,63].

#### Size and consistency of group composition

Knowing fellow group members was associated with trust and a feeling of safety which was described by students as crucial for participation in discussions and learning activities [48]. It is well known that trusting and safe environments promote student participation and engagement [47,48,63,67], but some have questioned whether it takes more time to develop trust in online groups compared with face-to-face groups [48]. This could be a partial explanation for our results showing that students prefer working in the same group over time. Other potential explanations offered in studies included in this review, were that working in the same group made the work process more predictable [47] and effective as the group got to know each other’s strengths and weaknesses [63]. The value of consistent groups is also apparent in the guidelines for Team-based learning, which recommends that students stay in the same group for as long as possible [73].

Further, our findings indicate that students prefer groups of 3–4 students. Supporting our results, a recent review on challenges and strategies for online group projects recommends an online group size of fewer than five students [13].

#### Organization

The findings in our scoping review suggest that organization of group work characterized by joint efforts, accountability, and responsibility among all group members, seems to foster a sense of belonging in the group as well as collaborative learning processes [45,47,52]. A strategy of working out solutions in the group, discussing each part, and taking responsibility for fellow students’ learning was found to promote understanding [47] and commitment to the group [52]. Another observed strategy of merely dividing tasks between group members was associated with lower performance [54]. These two strategies of group work have previously been discussed by Hammar Chiriac [8] as working as a group (collaborative) or in a group (cooperative). While collaborative learning is often referred to as ‘real group work’ as it involves reaching solutions through joint discussion and reflection [8], Donelan and Kear [13] advocate balancing cooperative and collaborative elements when creating assignments for online group work. They argue that cooperative learning may help alleviate unequal distribution of workload in online group work [13]. Unequal distribution of workload is a recurring challenge in group work [74], and it was also identified as a barrier in our scoping review. Using teaching methods that require students to be accountable for their contribution may be one way to mitigate this challenge. In health science education such teaching methods (e.g. Team-based learning [1,73], Case-based learning [75] and Problem-based learning [76]) are common because they use real case scenarios to prepare students for clinical work in healthcare teams. However, although many studies in our scoping review described using cases for discussions or assignments, only a few studies had used Team-based learning [45], Case-based learning [55], or other specific learning methods [45].

#### Challenges with scheduling group meetings

One of the most important barriers was challenges in scheduling group meetings [34,43,44,50,61,62,64,68]. Students from different countries, including the US, Canada, Australia and Indonesia, the UK and Somaliland, and Spain described that it was hard to find times to meet online due to everyone’s busy or different schedules as well as work obligations and personal responsibilities. Based on previous research, these are also common challenges in online group work in general [13]. However, the irregularity of shift work in the health sector may likely add to the challenge. Nevertheless, these findings indicate a need for further research into the cause and solutions for scheduling challenges.

#### Communication

Establishing and maintaining communication among students throughout online group work was essential in getting to know fellow group members, forming interpersonal relationships, and organising work processes. However, the students described establishing communication in online groups as challenging [34,46,64]. Lack of communication hindered the completion of the task and building learning relationships [34], and some had difficulties with contacting group members who did not participate actively [46]. While communication challenges can occur in all group work, not meeting in person may make it easier to delay or simply avoid answering, which could be described as ‘ghosting’ the group. Our findings revealed that social media and digital tools may facilitate communication [49,60,61]. Similarly, Donelan and Kear [13] point to the importance of selecting digital tools and means of synchronous communication. Despite its potential benefits to communication, the technical element is a common challenge in online group work [13] and was often reported as a barrier to communication and collaboration in our included studies. Students were frustrated with bad internet connection [34,49,59,63] which in addition to lag time [55] inhibited the flow of communication. Technical issues are recognized challenges in online group work and were also repeatedly reported in the studies included in Donelan and Kear’s review [13].

#### Stakeholder consultation

Two stakeholders read the results to increase the study validity and broaden our understanding of our findings (presentation of stakeholders’ background is found in Appendix 3). Both stakeholders found the results relevant. One stakeholder confirmed that unequal distribution of workload and dealing with ‘free-riders’ are challenging in online group work and that clear instructions and structure may facilitate the work process. It was also emphasized that including group members who keep their cameras off in online group work can be difficult. Our results were assessed as relatable and relevant in terms of implications for practitioners and future research.

#### Knowledge gaps and future research

Few of the included studies aimed to assess facilitators and barriers to online group work specifically [33,41,47,53,54,60]. Group work was mostly embedded in a pedagogical design or an integral part of a course and thus not addressed exclusively. This may be because it was not relevant to evaluate group work separately or because group work was considered too integrated in the total design to be separated. However, it may also suggest a knowledge gap in the literature, and a need for further research into online group work as a sole concept. Moreover, as this scoping review did not discriminate between different modes of online group work (e.g. synchronous, asynchronous, blended), additional investigation of facilitators and barriers specific to each mode of delivery could reveal important distinctions not observed in this review.

The findings in our scoping review mostly concerned students’ experiences, learning outcomes, and satisfaction, and only one article that reported on the educators’ perspective in addition to the students’ [67]. Although we did not include educators or faculty in our search words, our search strategy aimed to encompass higher education in general and should therefore have detected studies exploring the educators’ perspectives. The lack of such studies may reflect a limitation in our search strategy, but it may also indicate a knowledge gap. As the task of planning and designing health education ultimately falls on the teachers, exploring the topic from their point of view could provide valuable insight.

#### Strengths and limitations

The primary strength of this study is a thorough and broad search and screening process. The search strategy was developed in close collaboration with an experienced health science librarian to ensure methodological soundness. To increase reliability, all titles were screened, and data was charted by at least two researchers independently, and the researchers charting had many discussions throughout the process. One researcher screened all titles and charted data from all included studies to enhance consistency. Additionally, stakeholders were consulted at several stages of the process to improve the study validity.

The review has several limitations. By including only studies written in English or Scandinavian languages we may not have identified all relevant articles, and language bias may have been introduced. Also, seven of the 39 included articles were identified through hand searching, which could suggest weaknesses in our search strategy. We may have reached overly broad including studies regardless of whether online group work was the main or secondary focus, and regardless of the number of results per study related to online group work. However, group work is a common part of a course curriculum and thus often included in the course evaluation if only as a single item. We therefore assumed that we could lose information relevant to our research question if we excluded these studies. Despite adding a category for data not compatible with the presences in the framework, reviewing our studies through the lens of CoI may have caused us to overlook relevant data. Also, we are aware that our choices in categorization may have influenced our results and interpretation. Moreover, because we decided to use the CoI framework to guide our data analysis, we chose to summarize our data according to the framework rather than using a content analysis approach as proposed in the protocol. Consequently, we derived our main findings more subjectively, deciding to discuss only those facilitators and barriers we found to be of central importance, or integral to more than one element in the CoI Framework.

We chose to change the term digital (group work) used in the protocol, to online (group work) because the screening process revealed the latter to be more commonly used in the literature.

## Conclusion

This scoping review identified and summarized several potential facilitators and barriers to online group work in higher education within health sciences. Our findings suggest that creating smaller groups (3–4 students) that persist, and designing group work that foster joint responsibility may facilitate online student group work. Furthermore, making sure that students have access to necessary technological equipment and stable high-speed internet as well as providing support and structure for communication and scheduling meetings among the students may help alleviate potential barriers. We hope, by providing this overview to help researchers and educators within health sciences to navigate the literature on the topic and identify areas that need further research. However, this was an explorative study and further research is needed to investigate the different facilitators and barriers in more detail including the educators’ perspective.

## Supplementary Material

Supplemental Material

## Data Availability

Data charting schemes can be made available on request
