# Supplementary material for: Facilitators and barriers to online group work in higher education within health sciences – a scoping review
Source: Med Educ Online. 2024 Apr 12;29(1):2341508. doi: 10.1080/10872981.2024.2341508 (PMC11018044; doi:10.1080/10872981.2024.2341508)
Supplement: Supplemental Material [file ZMEO_A_2341508_SM1754.docx]

**Appendix 1 Full search strategy for the search performed 02.09.2022**

**Medline via Ovid**

| 1 | Education, Distance/ | 6139 |
| --- | --- | --- |
| 2 | (digital* OR online* OR virtual* OR blended OR hybrid OR distance learning OR distance education OR synchron* OR asynchron* OR decentra* OR e* learning OR electronic* learning OR compute* support*).tw,kw,kf. | 867839 |
| 3 | 1 OR 2 | 869504 |
| 4 | peer group/ | 23402 |
| 5 | (Collaborative learning OR group work OR small group* OR peer group* OR cooperative learning OR team learning OR tutorial group* OR peer learning OR group learning OR student group* OR group based OR team based OR peer assisted learning OR peer assisted OR discussion group).tw,kw,kf. | 44456 |
| 6 | 4 OR 5 | 65572 |
| 7 | education, graduate/ | 5946 |
| 8 | education, distance/ | 6139 |
| 9 | education, dental/ | 15520 |
| 10 | education, dental, graduate/ | 1931 |
| 11 | education, medical, graduate/ | 60235 |
| 12 | education, nursing, graduate/ | 32505 |
| 13 | education, pharmacy, graduate/ | 26534 |
| 14 | education, medical/ | 34261 |
| 15 | education, medical, undergraduate/ | 20457 |
| 16 | education, nursing/ | 3550 |
| 17 | education, nursing, baccalaureate/ | 8286 |
| 18 | education, nursing, diploma programs/ | 7131 |
| 19 | education, pharmacy/ | 1004 |
| 20 | students/ | 74747 |
| 21 | students, dental/ | 7012 |
| 22 | students, health occupations/ | 3286 |
| 23 | students, medical/ | 41260 |
| 24 | students, nursing/ | 28992 |
| 25 | students, pharmacy/ | 4190 |
| 26 | students, public health/ | 131 |
| 27 | or/7-26 | 322442 |
| 28 | (higher education* or health student* or graduate* or undergraduate* or postgraduate* or bachelor* or master* or tertiary education or postsecondary education or college or university or distance education or ((health science* or audiolog* or bioengineer* or biomedical engineering or cardiopulmonary technician* or chiropract* or dental* or dental hygien* or dental technician* or dentist* or dietetics* or dietitian* or emergency medical technician* or foot therap* or hearing care professiona* or medical* or midwi* or nurs* or nutritition* or occupational therap* or optician* or optometr* or orthoped* or paramedic* or perfusionis* or pharmacy or pharmacist* or physical therap* or physiotherap* or psycholog* or public health* or radiograph* or roentgenograph* or social educator* or health profession*) adj4 educat*) or ((health science* or audiolog* or bioengineer* or biomedical engineering or cardiopulmonary technician* or chiropract* or dental* or dental hygien* or dental technician* or dentist* or dietetics* or dietitian* emergency medical technician* or foot therap* or hearing care professiona* or medical* or midwi* or nurs* or nutritition* or occupational therap* or optician* or optometr* or orthoped* or paramedic* or perfusionis* or pharmacy or pharmacist* or physical therap* or physiotherap* or psycholog* or public health* or radiograph* or roentgenograph* or social educator* or health profession*) adj4 student*)).tw,kw,kf. | 831853 |
| 29 | 27 OR 28 | 978711 |
| 30 | audiologists/ | 187 |
| 31 | audiology/ | 2279 |
| 32 | Bioengineering/ | 2410 |
| 33 | biomedical engineering/ | 11358 |
| 34 | Chiropractic/ | 3421 |
| 35 | Dental Hygienists/ | 5859 |
| 36 | Dental Technicians/ | 2314 |
| 37 | dentistry/ | 36106 |
| 38 | Dentists/ | 19236 |
| 39 | dietetics/ | 8135 |
| 40 | emergency medical technicians/ | 5958 |
| 41 | health occupations/ | 8843 |
| 42 | Health personnel/ | 59230 |
| 43 | Medicine/ | 50868 |
| 44 | Midwifery/ | 20713 |
| 45 | Nurse Midwives/ | 7450 |
| 46 | nurses/ | 44110 |
| 47 | nursing education research/ | 9527 |
| 48 | nursing/ | 51750 |
| 49 | nutritional sciences/ | 11970 |
| 50 | Nutritionists/ | 1650 |
| 51 | Occupational Therapists/ | 583 |
| 52 | Occupational Therapy/ | 14442 |
| 53 | Optometrists/ | 195 |
| 54 | Optometry/ | 5682 |
| 55 | Orthopedics/ | 23236 |
| 56 | Pharmacists/ | 20617 |
| 57 | pharmacy technicians/ | 865 |
| 58 | pharmacy/ | 9520 |
| 59 | physical therapists/ | 2834 |
| 60 | physical therapy specialty/ | 2964 |
| 61 | Physicians/ | 99037 |
| 62 | Radiography/ | 325314 |
| 63 | students, dental/ | 7012 |
| 64 | students, health occupations/ | 3286 |
| 65 | students, medical/ | 41260 |
| 66 | students, nursing/ | 28992 |
| 67 | students, pharmacy/ | 4190 |
| 68 | education, dental, graduate/ | 15520 |
| 69 | education, dental/ | 1931 |
| 70 | education, medical, graduate/ | 60235 |
| 71 | education, medical, undergraduate/ | 32505 |
| 72 | education, medical/ | 26534 |
| 73 | education, nursing, baccalaureate/ | 34261 |
| 74 | education, nursing, diploma programs/ | 20457 |
| 75 | education, nursing, graduate/ | 3550 |
| 76 | education, nursing/ | 8286 |
| 77 | education, pharmacy, graduate/ | 7131 |
| 78 | education, pharmacy/ | 1004 |
| 79 | or/30-78 | 1030785 |
| 80 | (health occupation* or health personnel or health science* or audiolog* or bioengineer* or biomedical engineering or cardiopulmonary technician* or chiropract* or dental* or dental hygien* or dental technician* or dentist* or dietetics* or dietitian* emergency medical technician* or foot therap* or hearing care professiona* or medical* or midwi* or nurs* or nutritition* or occupational therap* or optician* or optometr* or orthoped* or paramedic* or perfusionis* or pharmacy or pharmacist* or physical therap* or physician* or physiotherap* or psycholog* or radiograph* or roentgenograph* or social educator* or ((health science* or audiolog* or bioengineer* or biomedical engineering or cardiopulmonary technician* or chiropract* or dental* or dental hygien* or dental technician* or dentist* or dietetics* or dietitian* emergency medical technician* or foot therap* or hearing care professiona* or medical* or midwi* or nurs* or nutritition* or occupational therap* or optician* or optometr* or orthoped* or paramedic* or perfusionis* or pharmacy or pharmacist* or physical therap* or physiotherap* or psycholog* or radiograph* or roentgenograph* or social educator* or health profession*) adj4 educat*) or ((health science* or audiolog* or bioengineer* or biomedical engineering or cardiopulmonary technician* or chiropract* or dental* or dental hygien* or dental technician* or dentist* or dietetics* or dietitian* emergency medical technician* or foot therap* or hearing care professiona* or medical* or midwi* or nurs* or nutritition* or occupational therap* or optician* or optometr* or orthoped* or paramedic* or perfusionis* or pharmacy or pharmacist* or physical therap* or physiotherap* or psycholog* or radiograph* or roentgenograph* or social educator* or health profession*) adj4 student*)).tw,kw,kf. | 3160853 |
| 81 | 79 OR 80 | 3687558 |
| 82 | 3 AND 6 AND 29 AND 81 | 1864 |
| 83 | limit 82 to ((danish or english or norwegian or swedish) and last 10 years) | 1448 |

**Embase via Ovid**

| 1 | e-learning/ | 4112 |
| --- | --- | --- |
| 2 | distance learning/ | 947 |
| 3 | virtual learning environment/ | 799 |
| 4 | 1 or 2 or 3 | 5490 |
| 5 | (digital* or online* or virtual* or blended or hybrid or distance learning or distance education or synchron* or asynchron* or decentra* or e* learning or electronic* learning or compute* support*).tw,kw,kf. | 1052278 |
| 6 | 4 or 5 | 1053383 |
| 7 | collaborative learning/ | 1486 |
| 8 | peer group/ | 28060 |
| 9 | 7 or 8 | 29510 |
| 10 | (Collaborative learning or group work or small group* or peer group* or cooperative learning or team learning or tutorial group* or peer learning or group learning or student group* or group based or team based or peer assisted learning or peer assisted or discussion group).tw,kw,kf. | 61343 |
| 11 | 9 or 10 | 87073 |
| 12 | college student/ | 19459 |
| 13 | graduate education/ | 163 |
| 14 | graduate student/ | 3507 |
| 15 | graduate/ | 7039 |
| 16 | masters education/ | 478 |
| 17 | postgraduate student/ | 8754 |
| 18 | student/ | 118439 |
| 19 | tertiary education/ | 2433 |
| 20 | undergraduate education/ | 486 |
| 21 | undergraduate student/ | 9034 |
| 22 | university student/ | 13400 |
| 23 | postgraduate education/ | 16978 |
| 24 | chiropractic education/ | 197 |
| 25 | dental education/ | 20045 |
| 26 | dental health education/ | 4589 |
| 27 | dietetics education/ | 132 |
| 28 | health education/ | 104220 |
| 29 | medical education/ | 241007 |
| 30 | midwifery education/ | 603 |
| 31 | nurse midwifery education/ | 62 |
| 32 | nursing education/ | 86929 |
| 33 | nutrition education/ | 6435 |
| 34 | occupational therapy education/ | 199 |
| 35 | paramedical education/ | 3863 |
| 36 | pharmacy education/ | 629 |
| 37 | physical therapy education/ | 221 |
| 38 | audiology student/ | 32 |
| 39 | baccalaureate nursing student/ | 73 |
| 40 | chiropractic student/ | 89 |
| 41 | dental hygiene student/ | 62 |
| 42 | dietetics student/ | 171 |
| 43 | graduate nursing student/ | 102 |
| 44 | health student/ | 1740 |
| 45 | medical student/ | 82615 |
| 46 | midwifery student/ | 408 |
| 47 | nursing student/ | 29948 |
| 48 | occupational therapy student/ | 245 |
| 49 | paramedical student/ | 414 |
| 50 | pharmacy student/ | 8617 |
| 51 | physical therapy student/ | 737 |
| 52 | public health student/ | 113 |
| 53 | or/12-52 | 687018 |
| 54 | (higher education* or health student* or graduate* or undergraduate* or postgraduate* or bachelor* or master* or tertiary education or postsecondary education or college or university or distance education or ((health science* or audiolog* or bioengineer* or biomedical engineering or cardiopulmonary technician* or chiropract* or dental* or dental hygien* or dental technician* or dentist* or dietetics* or dietitian* or emergency medical technician* or foot therap* or hearing care professiona* or medical* or midwi* or nurs* or nutritition* or occupational therap* or optician* or optometr* or orthoped* or paramedic* or perfusionis* or pharmacy or pharmacist* or physical therap* or physiotherap* or psycholog* or public health* or radiograph* or roentgenograph* or social educator* or health profession*) adj4 educat*) or ((health science* or audiolog* or bioengineer* or biomedical engineering or cardiopulmonary technician* or chiropract* or dental* or dental hygien* or dental technician* or dentist* or dietetics* or dietitian* emergency medical technician* or foot therap* or hearing care professiona* or medical* or midwi* or nurs* or nutritition* or occupational therap* or optician* or optometr* or orthoped* or paramedic* or perfusionis* or pharmacy or pharmacist* or physical therap* or physiotherap* or psycholog* or public health* or radiograph* or roentgenograph* or social educator* or health profession*) adj4 student*)).tw,kw,kf. | 1302275 |
| 55 | 53 or 54 | 1701221 |
| 56 | audiologist/ | 1146 |
| 57 | bioengineering/ | 20613 |
| 58 | biomedical engineering/ | 13707 |
| 59 | chiropractic/ | 4840 |
| 60 | chiropractor/ | 871 |
| 61 | dental auxiliary/ | 52 |
| 62 | dental hygienist/ | 704 |
| 63 | dental personnel/ | 152 |
| 64 | dental technician/ | 192 |
| 65 | dentist/ | 26361 |
| 66 | dentistry/ | 29460 |
| 67 | dietetics/ | 6039 |
| 68 | dietitian/ | 15299 |
| 69 | health care personnel/ | 208786 |
| 70 | health care/ | 123725 |
| 71 | medical personnel/ | 12608 |
| 72 | midwife/ | 28181 |
| 73 | nurse midwife/ | 7088 |
| 74 | nurse/ | 142304 |
| 75 | nursing/ | 205215 |
| 76 | occupational therapist/ | 7957 |
| 77 | occupational therapy/ | 24696 |
| 78 | optometrist/ | 1088 |
| 79 | optometry/ | 6481 |
| 80 | orthopedics/ | 23845 |
| 81 | paramedical personnel/ | 14642 |
| 82 | paramedical profession/ | 2033 |
| 83 | perfusionist/ | 274 |
| 84 | pharmacist/ | 85594 |
| 85 | pharmacy technician/ | 2543 |
| 86 | physician/ | 325150 |
| 87 | physiotherapist/ | 25445 |
| 88 | physiotherapy/ | 97884 |
| 89 | psychologist/ | 16280 |
| 90 | public health/ | 213188 |
| 91 | radiographer/ | 1047 |
| 92 | radiography/ | 199716 |
| 93 | rescue personnel/ | 8755 |
| 94 | chiropractic education/ | 197 |
| 95 | dental education/ | 20045 |
| 96 | dental health education/ | 4589 |
| 97 | dietetics education/ | 132 |
| 98 | health education/ | 104220 |
| 99 | medical education/ | 241007 |
| 100 | midwifery education/ | 603 |
| 101 | nurse midwifery education/ | 62 |
| 102 | nursing education/ | 86929 |
| 103 | nutrition education/ | 6435 |
| 104 | occupational therapy education/ | 199 |
| 105 | paramedical education/ | 3863 |
| 106 | pharmacy education/ | 629 |
| 107 | physical therapy education/ | 221 |
| 108 | audiology student/ | 32 |
| 109 | baccalaureate nursing student/ | 73 |
| 110 | chiropractic student/ | 89 |
| 111 | dental hygiene student/ | 62 |
| 112 | dietetics student/ | 171 |
| 113 | graduate nursing student/ | 102 |
| 114 | health student/ | 1740 |
| 115 | medical student/ | 82615 |
| 116 | midwifery student/ | 408 |
| 117 | nursing student/ | 29948 |
| 118 | occupational therapy student/ | 245 |
| 119 | paramedical student/ | 414 |
| 120 | pharmacy student/ | 8617 |
| 121 | physical therapy student/ | 737 |
| 122 | or/56-121 | 2078761 |
| 123 | (health occupation* or health personnel or health science* or audiolog* or bioengineer* or biomedical engineering or cardiopulmonary technician* or chiropract* or dental* or dental hygien* or dental technician* or dentist* or dietetics* or dietitian* emergency medical technician* or foot therap* or hearing care professiona* or medical* or midwi* or nurs* or nutritition* or occupational therap* or optician* or optometr* or orthoped* or paramedic* or perfusionis* or pharmacy or pharmacist* or physical therap* or physician* or physiotherap* or psycholog* or radiograph* or roentgenograph* or social educator* or ((health science* or audiolog* or bioengineer* or biomedical engineering or cardiopulmonary technician* or chiropract* or dental* or dental hygien* or dental technician* or dentist* or dietetics* or dietitian* emergency medical technician* or foot therap* or hearing care professiona* or medical* or midwi* or nurs* or nutritition* or occupational therap* or optician* or optometr* or orthoped* or paramedic* or perfusionis* or pharmacy or pharmacist* or physical therap* or physiotherap* or psycholog* or radiograph* or roentgenograph* or social educator* or health profession*) adj4 educat*) or ((health science* or audiolog* or bioengineer* or biomedical engineering or cardiopulmonary technician* or chiropract* or dental* or dental hygien* or dental technician* or dentist* or dietetics* or dietitian* emergency medical technician* or foot therap* or hearing care professiona* or medical* or midwi* or nurs* or nutritition* or occupational therap* or optician* or optometr* or orthoped* or paramedic* or perfusionis* or pharmacy or pharmacist* or physical therap* or physiotherap* or psycholog* or radiograph* or roentgenograph* or social educator* or health profession*) adj4 student*)).tw,kw,kf. | 4121056 |
| 124 | 122 or 123 | 5146486 |
| 125 | 6 and 11 and 55 and 124 | 3127 |
| 126 | limit 125 to ((danish or english or norwegian or swedish) and last 10 years) | 2461 |

**APA PsycINFO via Ovid**

| 1 | asynchronous learning/ | 68 |
| --- | --- | --- |
| 2 | blended learning/ | 706 |
| 3 | computer supported collaborative learning/ | 497 |
| 4 | Distance Education/ | 6887 |
| 5 | electronic learning/ | 2765 |
| 6 | virtual classrooms/ | 1033 |
| 7 | or/1-6 | 10697 |
| 8 | (digital* or online* or virtual* or blended or hybrid or distance learning or distance education or synchron* or asynchron* or decentra* or e* learning or electronic* learning or compute* support*).tw. | 246697 |
| 9 | 7 or 8 | 247365 |
| 10 | collaborative learning/ | 4033 |
| 11 | cooperative learning/ | 2868 |
| 12 | group discussion/ | 3877 |
| 13 | computer supported collaborative learning/ | 497 |
| 14 | team teaching method/ | 342 |
| 15 | peer tutoring/ | 1670 |
| 16 | or/10-15 | 12390 |
| 17 | (Collaborative learning or group work or small group* or peer group* or cooperative learning or team learning or tutorial group* or peer learning or group learning or student group* or group based or team based or peer assisted learning or peer assisted or discussion group).tw. | 45771 |
| 18 | 16 or 17 | 52701 |
| 19 | college graduates/ | 1444 |
| 20 | college students/ | 88711 |
| 21 | Graduate Education/ | 2910 |
| 22 | graduate students/ | 7825 |
| 23 | higher education/ | 20567 |
| 24 | postgraduate students/ | 1708 |
| 25 | postgraduate training/ | 1379 |
| 26 | students/ | 29516 |
| 27 | undergraduate education/ | 5682 |
| 28 | clinical psychology graduate training/ | 2687 |
| 29 | dental students/ | 317 |
| 30 | medical students/ | 14413 |
| 31 | Nursing Students/ | 6115 |
| 32 | graduate psychology education/ | 4800 |
| 33 | Dental Education/ | 218 |
| 34 | Health Education/ | 14354 |
| 35 | medical education/ | 19333 |
| 36 | nursing education/ | 7020 |
| 37 | or/19-36 | 202370 |
| 38 | (higher education* or health student* or graduate* or undergraduate* or postgraduate* or bachelor* or master* or tertiary education or postsecondary education or college or university or distance education or ((health science* or audiolog* or bioengineer* or biomedical engineering or cardiopulmonary technician* or chiropract* or dental* or dental hygien* or dental technician* or dentist* or dietetics* or dietitian* or emergency medical technician* or foot therap* or hearing care professiona* or medical* or midwi* or nurs* or nutritition* or occupational therap* or optician* or optometr* or orthoped* or paramedic* or perfusionis* or pharmacy or pharmacist* or physical therap* or physiotherap* or psycholog* or public health* or radiograph* or roentgenograph* or social educator* or health profession*) adj4 educat*) or ((health science* or audiolog* or bioengineer* or biomedical engineering or cardiopulmonary technician* or chiropract* or dental* or dental hygien* or dental technician* or dentist* or dietetics* or dietitian* emergency medical technician* or foot therap* or hearing care professiona* or medical* or midwi* or nurs* or nutritition* or occupational therap* or optician* or optometr* or orthoped* or paramedic* or perfusionis* or pharmacy or pharmacist* or physical therap* or physiotherap* or psycholog* or public health* or radiograph* or roentgenograph* or social educator* or health profession*) adj4 student*)).tw. | 564790 |
| 39 | 37 or 38 | 611275 |
| 40 | Dentistry/ | 486 |
| 41 | Dentists/ | 507 |
| 42 | health personnel/ | 19704 |
| 43 | Midwifery/ | 1646 |
| 44 | Nurses/ | 31031 |
| 45 | Nursing/ | 25566 |
| 46 | Occupational Therapists/ | 2513 |
| 47 | Occupational Therapy/ | 6890 |
| 48 | Optometrists/ | 127 |
| 49 | Optometry/ | 429 |
| 50 | Pharmacists/ | 1712 |
| 51 | physical therapists/ | 641 |
| 52 | psychologists/ | 23938 |
| 53 | roentgenography/ | 480 |
| 54 | physicians/ | 23703 |
| 55 | paramedics/ | 402 |
| 56 | paramedical sciences/ | 75 |
| 57 | clinical psychologists/ | 3151 |
| 58 | Dental Education/ | 218 |
| 59 | Health Education/ | 14354 |
| 60 | medical education/ | 19333 |
| 61 | nursing education/ | 7020 |
| 62 | dental students/ | 317 |
| 63 | medical students/ | 14413 |
| 64 | Nursing Students/ | 6115 |
| 65 | or/40-64 | 167497 |
| 66 | (health occupation* or health personnel or health science* or audiolog* or bioengineer* or biomedical engineering or cardiopulmonary technician* or chiropract* or dental* or dental hygien* or dental technician* or dentist* or dietetics* or dietitian* emergency medical technician* or foot therap* or hearing care professiona* or medical* or midwi* or nurs* or nutritition* or occupational therap* or optician* or optometr* or orthoped* or paramedic* or perfusionis* or pharmacy or pharmacist* or physical therap* or physician* or physiotherap* or psycholog* or radiograph* or roentgenograph* or social educator* or ((health science* or audiolog* or bioengineer* or biomedical engineering or cardiopulmonary technician* or chiropract* or dental* or dental hygien* or dental technician* or dentist* or dietetics* or dietitian* emergency medical technician* or foot therap* or hearing care professiona* or medical* or midwi* or nurs* or nutritition* or occupational therap* or optician* or optometr* or orthoped* or paramedic* or perfusionis* or pharmacy or pharmacist* or physical therap* or physiotherap* or psycholog* or radiograph* or roentgenograph* or social educator* or health profession*) adj4 educat*) or ((health science* or audiolog* or bioengineer* or biomedical engineering or cardiopulmonary technician* or chiropract* or dental* or dental hygien* or dental technician* or dentist* or dietetics* or dietitian* emergency medical technician* or foot therap* or hearing care professiona* or medical* or midwi* or nurs* or nutritition* or occupational therap* or optician* or optometr* or orthoped* or paramedic* or perfusionis* or pharmacy or pharmacist* or physical therap* or physiotherap* or psycholog* or radiograph* or roentgenograph* or social educator* or health profession*) adj4 student*)).tw. | 997323 |
| 67 | 65 or 66 | 1023672 |
| 68 | 9 and 18 and 39 and 67 | 707 |
| 69 | limit 68 to ((danish or english or norwegian or swedish) and last 10 years) | 454 |

**ERIC via EBSCOhost**

| S72 | S9 AND S17 AND S39 AND S71 Limiters - Date Published: 20120901-20220831; Language: Danish, English, Swedish | 288 |
| --- | --- | --- |
| S71 | S69 OR S70 | 138,22 |
| S70 | TI ( “health occupation*” OR “health personnel” OR “health science*” OR audiolog* OR bioengineer* OR “biomedical engineering” OR “cardiopulmonary technician*” OR chiropract* OR dental* OR “dental hygien*” OR “dental technician*” OR dentist* OR dietetics* OR dietitian* OR “emergency medical technician*” OR “foot therap*” OR “hearing care professiona*” OR medical* OR midwi* OR nurs* OR nutritition* OR “occupational therap*” OR optician* OR optometr* OR orthoped* OR paramedic* OR perfusionis* OR pharmacy OR pharmacist* OR “physical therap*” OR physiotherap* OR psycholog* OR radiograph* OR roentgenograph* OR “social educator*” OR ((“health science*” OR audiolog* OR bioengineer* OR “biomedical engineering” OR “cardiopulmonary technician*” OR chiropract* OR dental* OR “dental hygien*” OR “dental technician*” OR dentist* OR dietetics* OR dietitian* OR “emergency medical technician*” OR “foot therap*” OR “hearing care professiona*” OR medical* OR midwi* OR nurs* OR nutritition* OR “occupational therap*” OR optician* OR optometr* OR orthoped* OR paramedic* OR perfusionis* OR pharmacy OR pharmacist* OR “physical therap*” OR physiotherap* OR psycholog* OR ���public health” OR radiograph* OR roentgenograph* OR “social educator*” OR “health profession*”) N4 educat*) OR ((“health science*” OR audiolog* OR bioengineer* OR “biomedical engineering” OR “cardiopulmonary technician*” OR chiropract* OR dental* OR “dental hygien*” OR “dental technician*” OR dentist* OR dietetics* OR dietitian* OR “emergency medical technician*” OR “foot therap*” OR “hearing care professiona*” OR medical* OR midwi* OR nurs* OR nutritition* OR “occupational therap*” OR optician* OR optometr* OR orthoped* OR paramedic* OR perfusionis* OR pharmacy OR pharmacist* OR “physical therap*” OR physiotherap* OR psycholog* OR “public health” OR radiograph* OR roentgenograph* OR “social educator*” OR “health profession*”) N4 student*) ) OR AB ( “health occupation*” OR “health personnel” OR “health science*” OR audiolog* OR bioengineer* OR “biomedical engineering” OR “cardiopulmonary technician*” OR chiropract* OR dental* OR “dental hygien*” OR “dental technician*” OR dentist* OR dietetics* OR dietitian* OR “emergency medical technician*” OR “foot therap*” OR “hearing care professiona*” OR medical* OR midwi* OR nurs* OR nutritition* OR “occupational therap*” OR optician* OR optometr* OR orthoped* OR paramedic* OR perfusionis* OR pharmacy OR pharmacist* OR “physical therap*” OR physiotherap* OR psycholog* OR radiograph* OR roentgenograph* OR “social educator*” OR ((“health science*” OR audiolog* OR bioengineer* OR “biomedical engineering” OR “cardiopulmonary technician*” OR chiropract* OR dental* OR “dental hygien*” OR “dental technician*” OR dentist* OR dietetics* OR dietitian* OR “emergency medical technician*” OR “foot therap*” OR “hearing care professiona*” OR medical* OR midwi* OR nurs* OR nutritition* OR “occupational therap*” OR optician* OR optometr* OR orthoped* OR paramedic* OR perfusionis* OR pharmacy OR pharmacist* OR “physical therap*” OR physiotherap* OR psycholog* OR “public health” OR radiograph* OR roentgenograph* OR “social educator*” OR “health profession*”) N4 educat*) OR ((“health science*” OR audiolog* OR bioengineer* OR “biomedical engineering” OR “cardiopulmonary technician*” OR chiropract* OR dental* OR “dental hygien*” OR “dental technician*” OR dentist* OR dietetics* OR dietitian* OR “emergency medical technician*” OR “foot therap*” OR “hearing care professiona*” OR medical* OR midwi* OR nurs* OR nutritition* OR “occupational therap*” OR optician* OR optometr* OR orthoped* OR paramedic* OR perfusionis* OR pharmacy OR pharmacist* OR “physical therap*” OR physiotherap* OR psycholog* OR “public health” OR radiograph* OR roentgenograph* OR “social educator*” OR “health profession*”) N4 student*) ) | 112,122 |
| S69 | S40 OR S41 OR S42 OR S43 OR S44 OR S45 OR S46 OR S47 OR S48 OR S49 OR S50 OR S51 OR S52 OR S53 OR S54 OR S55 OR S56 OR S57 OR S58 OR S59 OR S60 OR S61 OR S62 OR S63 OR S64 OR S65 OR S66 OR S67 OR S68 | 57,598 |
| S68 | DE "Public Health" | 6,085 |
| S67 | DE "Nursing Students" | 1,303 |
| S66 | DE "Medical Students" | 4,728 |
| S65 | DE "Medical Education" | 9,874 |
| S64 | DE "Allied Health Occupations Education" | 5,109 |
| S63 | DE "Health Education" | 12,352 |
| S62 | DE "Graduate Medical Education" | 1,313 |
| S61 | DE "Nursing Education" | 5,455 |
| S60 | DE "Psychologists" | 2,548 |
| S59 | DE "Physicians" | 4,329 |
| S58 | DE "Physical Therapy" | 950 |
| S57 | DE "Pharmacy" | 733 |
| S56 | DE "Optometry" | 392 |
| S55 | DE "Occupational Therapy" | 1,185 |
| S54 | DE "Obstetrics" | 344 |
| S53 | DE "Nursing" | 2,184 |
| S52 | DE "Nurses" | 3,314 |
| S51 | DE "Medicine" | 2,376 |
| S50 | DE "Health Sciences" | 394 |
| S49 | DE "Health Personnel" | 3,192 |
| S48 | DE "Health Occupations" | 1,358 |
| S47 | DE "Emergency Medical Technicians" | 178 |
| S46 | DE "Dietetics" | 1,986 |
| S45 | DE "Dentistry" | 977 |
| S44 | DE "Clinical Psychology" | 1,073 |
| S43 | DE "Biomedicine" | 858 |
| S42 | DE "Audiology" | 490 |
| S41 | DE "Allied Health Personnel" | 2,851 |
| S40 | DE "Allied Health Occupations" | 1,19 |
| S39 | S37 OR S38 | 694,353 |
| S38 | TI ( “higher education*” OR “health student*” OR graduate* OR undergraduate* OR postgraduate* OR bachelor* OR master* OR “tertiary education” OR “postsecondary education” OR college OR university OR “distance education” OR ((“health science*” OR audiolog* OR bioengineer* OR “biomedical engineering” OR “cardiopulmonary technician*” OR chiropract* OR dental* OR “dental hygien*” OR “dental technician*” OR dentist* OR dietetics* OR dietitian* OR “emergency medical technician*” OR “foot therap*” OR “hearing care professiona*” OR medical* OR midwi* OR nurs* OR nutritition* OR “occupational therap*” OR optician* OR optometr* OR orthoped* OR paramedic* OR perfusionis* OR pharmacy OR pharmacist* OR “physical therap*” OR physiotherap* OR psycholog* OR “public health” OR radiograph* OR roentgenograph* OR “social educator*” OR “health profession*”) N4 educat*) OR ((“health science*” OR audiolog* OR bioengineer* OR “biomedical engineering” OR “cardiopulmonary technician*” OR chiropract* OR dental* OR “dental hygien*” OR “dental technician*” OR dentist* OR dietetics* OR dietitian* OR “emergency medical technician*” OR “foot therap*” OR “hearing care professiona*” OR medical* OR midwi* OR nurs* OR nutritition* OR “occupational therap*” OR optician* OR optometr* OR orthoped* OR paramedic* OR perfusionis* OR pharmacy OR pharmacist* OR “physical therap*” OR physiotherap* OR psycholog* OR “public health” OR radiograph* OR roentgenograph* OR “social educator*” OR “health profession*”) N4 student*) ) OR AB ( “higher education*” OR “health student*” OR graduate* OR undergraduate* OR postgraduate* OR bachelor* OR master* OR “tertiary education” OR “postsecondary education” OR college OR university OR “distance education” OR ((“health science*” OR audiolog* OR bioengineer* OR “biomedical engineering” OR “cardiopulmonary technician*” OR chiropract* OR dental* OR “dental hygien*” OR “dental technician*” OR dentist* OR dietetics* OR dietitian* OR “emergency medical technician*” OR “foot therap*” OR “hearing care professiona*” OR medical* OR midwi* OR nurs* OR nutritition* OR “occupational therap*” OR optician* OR optometr* OR orthoped* OR paramedic* OR perfusionis* OR pharmacy OR pharmacist* OR “physical therap*” OR physiotherap* OR psycholog* OR “public health” OR radiograph* OR roentgenograph* OR “social educator*” OR “health profession*”) N4 educat*) OR ((“health science*” OR audiolog* OR bioengineer* OR “biomedical engineering” OR “cardiopulmonary technician*” OR chiropract* OR dental* OR “dental hygien*” OR “dental technician*” OR dentist* OR dietetics* OR dietitian* OR “emergency medical technician*” OR “foot therap*” OR “hearing care professiona*” OR medical* OR midwi* OR nurs* OR nutritition* OR “occupational therap*” OR optician* OR optometr* OR orthoped* OR paramedic* OR perfusionis* OR pharmacy OR pharmacist* OR “physical therap*” OR physiotherap* OR psycholog* OR “public health” OR radiograph* OR roentgenograph* OR “social educator*” OR “health profession*”) N4 student*) ) | 495,598 |
| S37 | S18 OR S19 OR S20 OR S21 OR S22 OR S23 OR S24 OR S25 OR S26 OR S27 OR S28 OR S29 OR S30 OR S31 OR S32 OR S33 OR S34 OR S35 OR S36 | 559,665 |
| S36 | DE "Nursing Students" | 1,303 |
| S35 | DE "Medical Students" | 4,728 |
| S34 | DE "Medical Education" | 9,874 |
| S33 | DE "Allied Health Occupations Education" | 5,109 |
| S32 | DE "Health Education" | 12,352 |
| S31 | DE "Graduate Medical Education" | 1,313 |
| S30 | DE "Nursing Education" | 5,455 |
| S29 | DE "Postsecondary Education" | 184,026 |
| S28 | DE "Undergraduate Study" | 14,178 |
| S27 | DE "Undergraduate Students" | 41,469 |
| S26 | DE "Students" | 5,014 |
| S25 | DE "Masters Programs" | 4,5 |
| S24 | DE "Higher Education" | 486,476 |
| S23 | DE "Graduate Study" | 14,454 |
| S22 | DE "Graduate Students" | 21,479 |
| S21 | DE "College Seniors" | 1,572 |
| S20 | DE "College Freshmen" | 13,109 |
| S19 | DE "College Students" | 91,608 |
| S18 | DE "College Graduates" | 7,525 |
| S17 | S15 OR S16 | 54,773 |
| S16 | TI ( "Collaborative learning" OR "group work" OR "small group*" OR "peer group*" OR "cooperative learning" OR "team learning" OR "tutorial group*" OR "peer learning" OR "group learning" OR "student group*" OR "group based" OR "team based" OR "peer assisted learning" OR "peer assisted" OR "discussion group" ) OR AB ( "Collaborative learning" OR "group work" OR "small group*" OR "peer group*" OR "cooperative learning" OR "team learning" OR "tutorial group*" OR "peer learning" OR "group learning" OR "student group*" OR "group based" OR "team based" OR "peer assisted learning" OR "peer assisted" OR "discussion group" ) | 34,988 |
| S15 | S10 OR S11 OR S12 OR S13 OR S14 | 31,49 |
| S14 | DE "Peer Teaching" | 5,406 |
| S13 | DE "Peer Groups" | 2,755 |
| S12 | DE "Group Discussion" | 5,066 |
| S11 | DE "Discussion Groups" | 2,191 |
| S10 | DE "Cooperative Learning" | 17,928 |
| S9 | S7 OR S8 | 134,318 |
| S8 | TI ( digital* OR online* OR virtual* OR blended OR hybrid OR "distance learning" OR "distance education" OR synchron* OR asynchron* OR decentra* OR "e* learning" OR "electronic* learning" OR "compute* support*" ) OR AB ( digital* OR online* OR virtual* OR blended OR hybrid OR "distance learning" OR "distance education" OR synchron* OR asynchron* OR decentra* OR "e* learning" OR "electronic* learning" OR "compute* support*" ) | 126,59 |
| S7 | S1 OR S2 OR S3 OR S4 OR S5 OR S6 | 45,025 |
| S6 | DE "Virtual Classrooms" | 3,097 |
| S5 | DE "Online Courses" | 17,085 |
| S4 | DE "Electronic Learning" | 14,866 |
| S3 | DE "Electronic Classrooms" | 303 |
| S2 | DE "Distance Education" | 20,533 |
| S1 | DE "Blended Learning" | 5,746 |

**Education Source via EBSCOhost**

| S93 | S12 AND S19 AND S66 AND S92  Limiters - Published Date: 20120901-20220831; Language: Danish, English, Norwegian, Swedish | 488 |
| --- | --- | --- |
| S92 | S90 OR S91 | 291,468 |
| S91 | TI ( “health occupation*” OR “health personnel” OR “health science*” OR audiolog* OR bioengineer* OR “biomedical engineering” OR “cardiopulmonary technician*” OR chiropract* OR dental* OR “dental hygien*” OR “dental technician*” OR dentist* OR dietetics* OR dietitian* OR “emergency medical technician*” OR “foot therap*” OR “hearing care professiona*” OR medical* OR midwi* OR nurs* OR nutritition* OR “occupational therap*” OR optician* OR optometr* OR orthoped* OR paramedic* OR perfusionis* OR pharmacy OR pharmacist* OR “physical therap*” OR physiotherap* OR psycholog* OR radiograph* OR roentgenograph* OR “social educator*” OR ((“health science*” OR audiolog* OR bioengineer* OR “biomedical engineering” OR “cardiopulmonary technician*” OR chiropract* OR dental* OR “dental hygien*” OR “dental technician*” OR dentist* OR dietetics* OR dietitian* OR “emergency medical technician*” OR “foot therap*” OR “hearing care professiona*” OR medical* OR midwi* OR nurs* OR nutritition* OR “occupational therap*” OR optician* OR optometr* OR orthoped* OR paramedic* OR perfusionis* OR pharmacy OR pharmacist* OR “physical therap*” OR physiotherap* OR psycholog* OR “public health” OR radiograph* OR roentgenograph* OR “social educator*” OR “health profession*”) N4 educat*) OR ((“health science*” OR audiolog* OR bioengineer* OR “biomedical engineering” OR “cardiopulmonary technician*” OR chiropract* OR dental* OR “dental hygien*” OR “dental technician*” OR dentist* OR dietetics* OR dietitian* OR “emergency medical technician*” OR “foot therap*” OR “hearing care professiona*” OR medical* OR midwi* OR nurs* OR nutritition* OR “occupational therap*” OR optician* OR optometr* OR orthoped* OR paramedic* OR perfusionis* OR pharmacy OR pharmacist* OR “physical therap*” OR physiotherap* OR psycholog* OR “public health” OR radiograph* OR roentgenograph* OR “social educator*” OR “health profession*”) N4 student*) ) OR AB ( “health occupation*” OR “health personnel” OR “health science*” OR audiolog* OR bioengineer* OR “biomedical engineering” OR “cardiopulmonary technician*” OR chiropract* OR dental* OR “dental hygien*” OR “dental technician*” OR dentist* OR dietetics* OR dietitian* OR “emergency medical technician*” OR “foot therap*” OR “hearing care professiona*” OR medical* OR midwi* OR nurs* OR nutritition* OR “occupational therap*” OR optician* OR optometr* OR orthoped* OR paramedic* OR perfusionis* OR pharmacy OR pharmacist* OR “physical therap*” OR physiotherap* OR psycholog* OR radiograph* OR roentgenograph* OR “social educator*” OR ((“health science*” OR audiolog* OR bioengineer* OR “biomedical engineering” OR “cardiopulmonary technician*” OR chiropract* OR dental* OR “dental hygien*” OR “dental technician*” OR dentist* OR dietetics* OR dietitian* OR “emergency medical technician*” OR “foot therap*” OR “hearing care professiona*” OR medical* OR midwi* OR nurs* OR nutritition* OR “occupational therap*” OR optician* OR optometr* OR orthoped* OR paramedic* OR perfusionis* OR pharmacy OR pharmacist* OR “physical therap*” OR physiotherap* OR psycholog* OR “public health” OR radiograph* OR roentgenograph* OR “social educator*” OR “health profession*”) N4 educat*) OR ((“health science*” OR audiolog* OR bioengineer* OR “biomedical engineering” OR “cardiopulmonary technician*” OR chiropract* OR dental* OR “dental hygien*” OR “dental technician*” OR dentist* OR dietetics* OR dietitian* OR “emergency medical technician*” OR “foot therap*” OR “hearing care professiona*” OR medical* OR midwi* OR nurs* OR nutritition* OR “occupational therap*” OR optician* OR optometr* OR orthoped* OR paramedic* OR perfusionis* OR pharmacy OR pharmacist* OR “physical therap*” OR physiotherap* OR psycholog* OR “public health” OR radiograph* OR roentgenograph* OR “social educator*” OR “health profession*”) N4 student*) ) | 284,85 |
| S90 | S67 OR S68 OR S69 OR S70 OR S71 OR S72 OR S73 OR S74 OR S75 OR S76 OR S77 OR S78 OR S79 OR S80 OR S81 OR S82 OR S83 OR S84 OR S85 OR S86 OR S87 OR S88 OR S89 | 40,836 |
| S89 | DE "Pharmacy students" | 2,218 |
| S88 | DE "Occupational therapy students" | 57 |
| S87 | DE "Nursing students" | 7,951 |
| S86 | DE "Medical students" | 9,159 |
| S85 | DE "Health occupations students" | 3,097 |
| S84 | DE "Nutrition students" | 9 |
| S83 | DE "Dental students" | 1,972 |
| S82 | DE "Chiropractic students" | 6 |
| S81 | DE "Graduate medical education" | 265 |
| S80 | DE "Graduate psychology education" | 279 |
| S79 | DE "Education of physicians" | 268 |
| S78 | DE "Physical therapy education" | 141 |
| S77 | DE "Pharmacy education" | 2,737 |
| S76 | DE "Nutrition education (Higher)" | 105 |
| S75 | DE "Midwifery education" | 316 |
| S74 | DE "Medical education" | 13,907 |
| S73 | DE "Health education (Higher)" | 11 |
| S72 | DE "Dental education" | 2,947 |
| S71 | DE "Clinical psychology education in graduate schools" | 21 |
| S70 | DE "Chiropractic education" | 11 |
| S69 | DE "Baccalaureate nursing education" | 1,431 |
| S68 | DE "Clinical psychology" | 3,109 |
| S67 | DE "Occupational therapists" | 305 |
| S66 | S64 OR S65 | 975,772 |
| S65 | TI ( “higher education*” OR “health student*” OR graduate* OR undergraduate* OR postgraduate* OR bachelor* OR master* OR “tertiary education” OR “postsecondary education” OR college OR university OR “distance education” OR ((“health science*” OR audiolog* OR bioengineer* OR “biomedical engineering” OR “cardiopulmonary technician*” OR chiropract* OR dental* OR “dental hygien*” OR “dental technician*” OR dentist* OR dietetics* OR dietitian* OR “emergency medical technician*” OR “foot therap*” OR “hearing care professiona*” OR medical* OR midwi* OR nurs* OR nutritition* OR “occupational therap*” OR optician* OR optometr* OR orthoped* OR paramedic* OR perfusionis* OR pharmacy OR pharmacist* OR “physical therap*” OR physiotherap* OR psycholog* OR “public health” OR radiograph* OR roentgenograph* OR “social educator*” OR “health profession*”) N4 educat*) OR ((“health science*” OR audiolog* OR bioengineer* OR “biomedical engineering” OR “cardiopulmonary technician*” OR chiropract* OR dental* OR “dental hygien*” OR “dental technician*” OR dentist* OR dietetics* OR dietitian* OR “emergency medical technician*” OR “foot therap*” OR “hearing care professiona*” OR medical* OR midwi* OR nurs* OR nutritition* OR “occupational therap*” OR optician* OR optometr* OR orthoped* OR paramedic* OR perfusionis* OR pharmacy OR pharmacist* OR “physical therap*” OR physiotherap* OR psycholog* OR “public health” OR radiograph* OR roentgenograph* OR “social educator*” OR “health profession*”) N4 student*) ) OR AB ( “higher education*” OR “health student*” OR graduate* OR undergraduate* OR postgraduate* OR bachelor* OR master* OR “tertiary education” OR “postsecondary education” OR college OR university OR “distance education” OR ((“health science*” OR audiolog* OR bioengineer* OR “biomedical engineering” OR “cardiopulmonary technician*” OR chiropract* OR dental* OR “dental hygien*” OR “dental technician*” OR dentist* OR dietetics* OR dietitian* OR “emergency medical technician*” OR “foot therap*” OR “hearing care professiona*” OR medical* OR midwi* OR nurs* OR nutritition* OR “occupational therap*” OR optician* OR optometr* OR orthoped* OR paramedic* OR perfusionis* OR pharmacy OR pharmacist* OR “physical therap*” OR physiotherap* OR psycholog* OR “public health” OR radiograph* OR roentgenograph* OR “social educator*” OR “health profession*”) N4 educat*) OR ((“health science*” OR audiolog* OR bioengineer* OR “biomedical engineering” OR “cardiopulmonary technician*” OR chiropract* OR dental* OR “dental hygien*” OR “dental technician*” OR dentist* OR dietetics* OR dietitian* OR “emergency medical technician*” OR “foot therap*” OR “hearing care professiona*” OR medical* OR midwi* OR nurs* OR nutritition* OR “occupational therap*” OR optician* OR optometr* OR orthoped* OR paramedic* OR perfusionis* OR pharmacy OR pharmacist* OR “physical therap*” OR physiotherap* OR psycholog* OR “public health” OR radiograph* OR roentgenograph* OR “social educator*” OR “health profession*”) N4 student*) ) | 925,688 |
| S64 | S20 OR S21 OR S22 OR S23 OR S24 OR S25 OR S26 OR S27 OR S28 OR S29 OR S30 OR S31 OR S32 OR S33 OR S34 OR S35 OR S36 OR S37 OR S38 OR S39 OR S40 OR S41 OR S42 OR S43 OR S44 OR S45 OR S46 OR S47 OR S48 OR S49 OR S50 OR S51 OR S52 OR S53 OR S54 OR S55 OR S56 OR S57 OR S58 OR S59 OR S60 OR S61 OR S62 OR S63 | 270,814 |
| S63 | DE "Online education" | 22,008 |
| S62 | DE "Distance education graduates" | 5 |
| S61 | DE "Distance higher education" | 173 |
| S60 | DE "Distance education students" | 1,308 |
| S59 | DE "Pharmacy students" | 2,218 |
| S58 | DE "Occupational therapy students" | 57 |
| S57 | DE "Nursing students" | 7,951 |
| S56 | DE "Medical students" | 9,159 |
| S55 | DE "Health occupations students" | 3,097 |
| S54 | DE "Nutrition students" | 9 |
| S53 | DE "Dental students" | 1,972 |
| S52 | DE "Chiropractic students" | 6 |
| S51 | DE "Physical therapy education" | 141 |
| S50 | DE "Public health education (Graduate)" | 21 |
| S49 | DE "Public health education (Higher)" | 26 |
| S48 | DE "Optometry education" | 151 |
| S47 | DE "Graduate nursing education" | 1,121 |
| S46 | DE "Graduate medical education" | 265 |
| S45 | DE "Graduate psychology education" | 279 |
| S44 | DE "Education of physicians" | 268 |
| S43 | DE "Physical therapy education" | 141 |
| S42 | DE "Pharmacy education" | 2,737 |
| S41 | DE "Nutrition education (Higher)" | 105 |
| S40 | DE "Midwifery education" | 316 |
| S39 | DE "Medical education" | 13,907 |
| S38 | DE "Health education (Higher)" | 11 |
| S37 | DE "Dental education" | 2,947 |
| S36 | DE "Clinical psychology education in graduate schools" | 21 |
| S35 | DE "Chiropractic education" | 11 |
| S34 | DE "Baccalaureate nursing education" | 1,431 |
| S33 | DE "Audiology education" | 60 |
| S32 | DE "Undergraduates" | 12,596 |
| S31 | DE "Undergraduate programs" | 2,407 |
| S30 | DE "Postsecondary education" | 9,012 |
| S29 | DE "Masters programs (Higher education)" | 892 |
| S28 | DE "Higher education" | 133,62 |
| S27 | DE "Graduate students" | 9,693 |
| S26 | DE "Graduate education" | 12,597 |
| S25 | DE "College students" | 52,046 |
| S24 | DE "College sophomores" | 267 |
| S23 | DE "College seniors" | 186 |
| S22 | DE "College juniors" | 46 |
| S21 | DE "College graduates" | 5,705 |
| S20 | DE "College freshmen | 3,552 |
| S19 | S17 OR S18 | 46,831 |
| S18 | TI ( "Collaborative learning" OR "group work" OR "small group*" OR "peer group*" OR "cooperative learning" OR "team learning" OR "tutorial group*" OR "peer learning" OR "group learning" OR "student group*" OR "group based" OR "team based" OR "peer assisted learning" OR "peer assisted" OR "discussion group*" ) OR AB ( "Collaborative learning" OR "group work" OR "small group*" OR "peer group*" OR "cooperative learning" OR "team learning" OR "tutorial group*" OR "peer learning" OR "group learning" OR "student group*" OR "group based" OR "team based" OR "peer assisted learning" OR "peer assisted" OR "discussion group*" ) | 36,552 |
| S17 | S13 OR S14 OR S15 OR S16 | 18,748 |
| S16 | DE "Peer teaching" | 1,85 |
| S15 | DE "Group work in education" | 9,909 |
| S14 | DE "Team learning approach in education" | 2,294 |
| S13 | DE "Collaborative learning" | 6,803 |
| S12 | S10 OR S11 | 291,914 |
| S11 | TI ( digital* OR online* OR virtual* OR blended OR hybrid OR "distance learning" OR "distance education" OR synchron* OR asynchron* OR decentra* OR "e* learning" OR "electronic* learning" OR "compute* support*" ) OR AB ( digital* OR online* OR virtual* OR blended OR hybrid OR "distance learning" OR "distance education" OR synchron* OR asynchron* OR decentra* OR "e* learning" OR "electronic* learning" OR "compute* support*" ) | 283,954 |
| S10 | S1 OR S2 OR S3 OR S4 OR S5 OR S6 OR S7 OR S8 OR S9 | 48,143 |
| S9 | DE "Distance education students" | 1,308 |
| S8 | DE "Asynchronous learning" | 554 |
| S7 | DE "Distance education graduates" | 5 |
| S6 | DE "Distance higher education" | 173 |
| S5 | DE "Distance education" | 25,096 |
| S4 | DE "Virtual classrooms" | 1,691 |
| S3 | DE "Blended learning" | 4,543 |
| S2 | DE "Online education" | 22,008 |
| S1 | DE "Digital learning" | 828 |

**CINAHL via EBSCOhost**

| S108 | S3 AND S6 AND S45 AND S107  Limiters - Published Date: 20120901-20220831; Language: Danish, English, Norwegian, Swedish | 678 |
| --- | --- | --- |
| S107 | S105 OR S106 | 1,679,898 |
| S106 | TI ( “health occupation*” OR “health personnel” OR “health science*” OR audiolog* OR bioengineer* OR “biomedical engineering” OR “cardiopulmonary technician*” OR chiropract* OR dental* OR “dental hygien*” OR “dental technician*” OR dentist* OR dietetics* OR dietitian* OR “emergency medical technician*” OR “foot therap*” OR “hearing care professiona*” OR medical* OR midwi* OR nurs* OR nutritition* OR “occupational therap*” OR optician* OR optometr* OR orthoped* OR paramedic* OR perfusionis* OR pharmacy OR pharmacist* OR “physical therap*” OR physiotherap* OR psycholog* OR radiograph* OR roentgenograph* OR “social educator*” OR ((“health science*” OR audiolog* OR bioengineer* OR “biomedical engineering” OR “cardiopulmonary technician*” OR chiropract* OR dental* OR “dental hygien*” OR “dental technician*” OR dentist* OR dietetics* OR dietitian* OR “emergency medical technician*” OR “foot therap*” OR “hearing care professiona*” OR medical* OR midwi* OR nurs* OR nutritition* OR “occupational therap*” OR optician* OR optometr* OR orthoped* OR paramedic* OR perfusionis* OR pharmacy OR pharmacist* OR “physical therap*” OR physiotherap* OR psycholog* OR “public health” OR radiograph* OR roentgenograph* OR “social educator*” OR “health profession*”) N4 educat*) OR ((“health science*” OR audiolog* OR bioengineer* OR “biomedical engineering” OR “cardiopulmonary technician*” OR chiropract* OR dental* OR “dental hygien*” OR “dental technician*” OR dentist* OR dietetics* OR dietitian* OR “emergency medical technician*” OR “foot therap*” OR “hearing care professiona*” OR medical* OR midwi* OR nurs* OR nutritition* OR “occupational therap*” OR optician* OR optometr* OR orthoped* OR paramedic* OR perfusionis* OR pharmacy OR pharmacist* OR “physical therap*” OR physiotherap* OR psycholog* OR “public health” OR radiograph* OR roentgenograph* OR “social educator*” OR “health profession*”) N4 student*) ) OR AB ( “health occupation*” OR “health personnel” OR “health science*” OR audiolog* OR bioengineer* OR “biomedical engineering” OR “cardiopulmonary technician*” OR chiropract* OR dental* OR “dental hygien*” OR “dental technician*” OR dentist* OR dietetics* OR dietitian* OR “emergency medical technician*” OR “foot therap*” OR “hearing care professiona*” OR medical* OR midwi* OR nurs* OR nutritition* OR “occupational therap*” OR optician* OR optometr* OR orthoped* OR paramedic* OR perfusionis* OR pharmacy OR pharmacist* OR “physical therap*” OR physiotherap* OR psycholog* OR radiograph* OR roentgenograph* OR “social educator*” OR ((“health science*” OR audiolog* OR bioengineer* OR “biomedical engineering” OR “cardiopulmonary technician*” OR chiropract* OR dental* OR “dental hygien*” OR “dental technician*” OR dentist* OR dietetics* OR dietitian* OR “emergency medical technician*” OR “foot therap*” OR “hearing care professiona*” OR medical* OR midwi* OR nurs* OR nutritition* OR “occupational therap*” OR optician* OR optometr* OR orthoped* OR paramedic* OR perfusionis* OR pharmacy OR pharmacist* OR “physical therap*” OR physiotherap* OR psycholog* OR “public health” OR radiograph* OR roentgenograph* OR “social educator*” OR “health profession*”) N4 educat*) OR ((“health science*” OR audiolog* OR bioengineer* OR “biomedical engineering” OR “cardiopulmonary technician*” OR chiropract* OR dental* OR “dental hygien*” OR “dental technician*” OR dentist* OR dietetics* OR dietitian* OR “emergency medical technician*” OR “foot therap*” OR “hearing care professiona*” OR medical* OR midwi* OR nurs* OR nutritition* OR “occupational therap*” OR optician* OR optometr* OR orthoped* OR paramedic* OR perfusionis* OR pharmacy OR pharmacist* OR “physical therap*” OR physiotherap* OR psycholog* OR “public health” OR radiograph* OR roentgenograph* OR “social educator*” OR “health profession*”) N4 student*) ) | 1,414,297 |
| S105 | S46 OR S47 OR S48 OR S49 OR S50 OR S51 OR S52 OR S53 OR S54 OR S55 OR S56 OR S57 OR S58 OR S59 OR S60 OR S61 OR S62 OR S63 OR S64 OR S65 OR S66 OR S67 OR S68 OR S69 OR S70 OR S71 OR S72 OR S73 OR S74 OR S75 OR S76 OR S77 OR S78 OR S79 OR S80 OR S81 OR S82 OR S83 OR S84 OR S85 OR S86 OR S87 OR S88 OR S89 OR S90 OR S91 OR S92 OR S93 OR S94 OR S95 OR S96 OR S97 OR S98 OR S99 OR S100 OR S101 OR S102 OR S103 OR S104 | 533,876 |
| S104 | (MH "Students, Physical Therapy") | 2,515 |
| S103 | (MH "Students, Pharmacy") | 1,578 |
| S102 | (MH "Students, Occupational Therapy") | 2,08 |
| S101 | (MH "Students, Midwifery") | 2,375 |
| S100 | (MH "Students, Medical") | 18,645 |
| S99 | (MH "Students, Dietetics") | 386 |
| S98 | (MH "Students, Dental Hygiene") | 715 |
| S97 | (MH "Students, Dental") | 2,996 |
| S96 | (MH "Students, Chiropractic") | 724 |
| S95 | (MH "Students, Audiology") | 623 |
| S94 | (MH "Education, Pharmacy Technicians") | 56 |
| S93 | (MH "Education, Pharmacy") | 2,065 |
| S92 | (MH "Education, Occupational Therapy") | 3,905 |
| S91 | (MH "Education, Nurse Midwifery") | 713 |
| S90 | (MH "Education, Nursing, Masters") | 2,357 |
| S89 | (MH "Education, Nursing, Graduate") | 3,352 |
| S88 | (MH "Education, Nursing, Diploma Programs") | 693 |
| S87 | (MH "Education, Nursing, Baccalaureate") | 10,3 |
| S86 | (MH "Education, Nursing") | 46,482 |
| S85 | (MH "Education, Midwifery") | 3,714 |
| S84 | (MH "Education, Medical") | 34,42 |
| S83 | (MH "Education, Health Sciences") | 1,976 |
| S82 | (MH "Education, Emergency Medical Services") | 1,668 |
| S81 | (MH "Education, Dietetics") | 803 |
| S80 | (MH "Education, Dental Hygiene") | 1,145 |
| S79 | (MH "Education, Dental") | 4,231 |
| S78 | (MH "Education, Chiropractic") | 1,783 |
| S77 | (MH "Education, Audiology") | 994 |
| S76 | (MH "Psychology, Clinical") | 1,072 |
| S75 | (MH "Psychologists") | 4,843 |
| S74 | (MH "Physicians") | 65,801 |
| S73 | (MH "Physical Therapy") | 37,721 |
| S72 | (MH "Physical Therapists") | 13,911 |
| S71 | (MH "Pharmacy Technicians") | 989 |
| S70 | (MH "Pharmacy and Pharmacology") | 10,121 |
| S69 | (MH "Pharmacists") | 18,132 |
| S68 | (MH "Orthopedics") | 13,349 |
| S67 | (MH "Optometry") | 3,53 |
| S66 | (MH "Optometrists") | 549 |
| S65 | (MH "Occupational Therapy") | 24,029 |
| S64 | (MH "Occupational Therapists") | 10,189 |
| S63 | (MH "Nurses") | 68,498 |
| S62 | (MH "Midwives") | 14,037 |
| S61 | (MH "Midwifery") | 19,136 |
| S60 | (MH "Medicine") | 7,956 |
| S59 | (MH "Health Personnel") | 49,136 |
| S58 | (MH "Emergency Medical Technicians") | 12,91 |
| S57 | (MH "Dietitians") | 5,809 |
| S56 | (MH "Dentists") | 11,134 |
| S55 | (MH "Dentistry") | 13,704 |
| S54 | (MH "Dental Technicians") | 454 |
| S53 | (MH "Dental Hygienists") | 7,946 |
| S52 | (MH "Dental Hygiene") | 5,369 |
| S51 | (MH "Dental Auxiliaries") | 1,314 |
| S50 | (MH "Chiropractors") | 5,479 |
| S49 | (MH "Chiropractic") | 17,336 |
| S48 | (MH "Cardiopulmonary Technicians") | 150 |
| S47 | (MH "Biomedical Engineering") | 2,407 |
| S46 | (MH "Audiologists") | 3,72 |
| S45 | S43 OR S44 | 480,115 |
| S44 | TI ( “higher education*” OR “health student*” OR graduate* OR undergraduate* OR postgraduate* OR bachelor* OR master* OR “tertiary education” OR “postsecondary education” OR college OR university OR “distance education” OR ((“health science*” OR audiolog* OR bioengineer* OR “biomedical engineering” OR “cardiopulmonary technician*” OR chiropract* OR dental* OR “dental hygien*” OR “dental technician*” OR dentist* OR dietetics* OR dietitian* OR “emergency medical technician*” OR “foot therap*” OR “hearing care professiona*” OR medical* OR midwi* OR nurs* OR nutritition* OR “occupational therap*” OR optician* OR optometr* OR orthoped* OR paramedic* OR perfusionis* OR pharmacy OR pharmacist* OR “physical therap*” OR physiotherap* OR psycholog* OR “public health” OR radiograph* OR roentgenograph* OR “social educator*” OR “health profession*”) N4 educat*) OR ((“health science*” OR audiolog* OR bioengineer* OR “biomedical engineering” OR “cardiopulmonary technician*” OR chiropract* OR dental* OR “dental hygien*” OR “dental technician*” OR dentist* OR dietetics* OR dietitian* OR “emergency medical technician*” OR “foot therap*” OR “hearing care professiona*” OR medical* OR midwi* OR nurs* OR nutritition* OR “occupational therap*” OR optician* OR optometr* OR orthoped* OR paramedic* OR perfusionis* OR pharmacy OR pharmacist* OR “physical therap*” OR physiotherap* OR psycholog* OR “public health” OR radiograph* OR roentgenograph* OR “social educator*” OR “health profession*”) N4 student*) ) OR AB ( “higher education*” OR “health student*” OR graduate* OR undergraduate* OR postgraduate* OR bachelor* OR master* OR “tertiary education” OR “postsecondary education” OR college OR university OR “distance education” OR ((“health science*” OR audiolog* OR bioengineer* OR “biomedical engineering” OR “cardiopulmonary technician*” OR chiropract* OR dental* OR “dental hygien*” OR “dental technician*” OR dentist* OR dietetics* OR dietitian* OR “emergency medical technician*” OR “foot therap*” OR “hearing care professiona*” OR medical* OR midwi* OR nurs* OR nutritition* OR “occupational therap*” OR optician* OR optometr* OR orthoped* OR paramedic* OR perfusionis* OR pharmacy OR pharmacist* OR “physical therap*” OR physiotherap* OR psycholog* OR “public health” OR radiograph* OR roentgenograph* OR “social educator*” OR “health profession*”) N4 educat*) OR ((“health science*” OR audiolog* OR bioengineer* OR “biomedical engineering” OR “cardiopulmonary technician*” OR chiropract* OR dental* OR “dental hygien*” OR “dental technician*” OR dentist* OR dietetics* OR dietitian* OR “emergency medical technician*” OR “foot therap*” OR “hearing care professiona*” OR medical* OR midwi* OR nurs* OR nutritition* OR “occupational therap*” OR optician* OR optometr* OR orthoped* OR paramedic* OR perfusionis* OR pharmacy OR pharmacist* OR “physical therap*” OR physiotherap* OR psycholog* OR “public health” OR radiograph* OR roentgenograph* OR “social educator*” OR “health profession*”) N4 student*) ) | 401,743 |
| S43 | S7 OR S8 OR S9 OR S10 OR S11 OR S12 OR S13 OR S14 OR S15 OR S16 OR S17 OR S18 OR S19 OR S20 OR S21 OR S22 OR S23 OR S24 OR S25 OR S26 OR S27 OR S28 OR S29 OR S30 OR S31 OR S32 OR S33 OR S34 OR S35 OR S36 OR S37 OR S38 OR S39 OR S40 OR S41 OR S42 | 178,88 |
| S42 | (MH "Online Education") | 2,537 |
| S41 | (MH "Students, Physical Therapy") | 2,515 |
| S40 | (MH "Students, Pharmacy") | 1,578 |
| S39 | (MH "Students, Occupational Therapy") | 2,08 |
| S38 | (MH "Students, Midwifery") | 2,375 |
| S37 | (MH "Students, Medical") | 18,645 |
| S36 | (MH "Students, Dietetics") | 386 |
| S35 | (MH "Students, Dental Hygiene") | 715 |
| S34 | (MH "Students, Dental") | 2,996 |
| S33 | (MH "Students, Chiropractic") | 724 |
| S32 | (MH "Students, Audiology") | 623 |
| S31 | (MH "Education, Pharmacy Technicians") | 56 |
| S30 | (MH "Education, Pharmacy") | 2,065 |
| S29 | (MH "Education, Occupational Therapy") | 3,905 |
| S28 | (MH "Education, Nurse Midwifery") | 713 |
| S27 | (MH "Education, Nursing, Masters") | 2,357 |
| S26 | (MH "Education, Nursing, Graduate") | 3,352 |
| S25 | (MH "Education, Nursing, Diploma Programs") | 693 |
| S24 | (MH "Education, Nursing, Baccalaureate") | 10,3 |
| S23 | (MH "Education, Nursing") | 46,482 |
| S22 | (MH "Education, Midwifery") | 3,714 |
| S21 | (MH "Education, Medical") | 34,42 |
| S20 | (MH "Education, Health Sciences") | 1,976 |
| S19 | (MH "Education, Emergency Medical Services") | 1,668 |
| S18 | (MH "Education, Dietetics") | 803 |
| S17 | (MH "Education, Dental Hygiene") | 1,145 |
| S16 | (MH "Education, Dental" | 4,231 |
| S15 | (MH "Education, Chiropractic") | 1,783 |
| S14 | (MH "Education, Audiology") | 994 |
| S13 | (MH "Students, Graduate") | 2,683 |
| S12 | (MH "Students, Undergraduate") | 9,1 |
| S11 | (MH "Students, College") | 27,842 |
| S10 | (MH "Education, Baccalaureate") | 1,973 |
| S9 | (MH "College Graduates") | 473 |
| S8 | (MH "Education, Masters") | 2,426 |
| S7 | (MH "Education, Graduate") | 3,035 |
| S6 | S4 OR S5 | 34,831 |
| S5 | TI ( "Collaborative learning" OR "group work" OR "small group*" OR "peer group*" OR "cooperative learning" OR "team learning" OR "tutorial group*" OR "peer learning" OR "group learning" OR "student group*" OR "group based" OR "team based" OR "peer assisted learning" OR "peer assisted" OR "discussion group*" ) OR AB ( "Collaborative learning" OR "group work" OR "small group*" OR "peer group*" OR "cooperative learning" OR "team learning" OR "tutorial group*" OR "peer learning" OR "group learning" OR "student group*" OR "group based" OR "team based" OR "peer assisted learning" OR "peer assisted" OR "discussion group*" ) | 20,683 |
| S4 | (MH "Peer Group") | 15,439 |
| S3 | S1 OR S2 | 215,895 |
| S2 | TI ( digital* OR online* OR virtual* OR blended OR hybrid OR "distance learning" OR "distance education" OR synchron* OR asynchron* OR decentra* OR "e* learning" OR "electronic* learning" OR "compute* support*" ) OR AB ( digital* OR online* OR virtual* OR blended OR hybrid OR "distance learning" OR "distance education" OR synchron* OR asynchron* OR decentra* OR "e* learning" OR "electronic* learning" OR "compute* support*" ) | 215,089 |
| S1 | (MH "Online Education") | 2,537 |

**Scopus**

| limited to 2012-2022 and english language | 358 |
| --- | --- |
| ( (TITLE ( ( {health occupation} )  OR  ( {health occupations} )  OR  ( {health personnel} )  OR  ( {health science} )  OR  ( {health sciences} )  OR  audiolog*  OR  bioengineer*  OR  ( {biomedical engineering} )  OR  ( {cardiopulmonary technician} )  OR  ( {cardiopulmonary technicians} )  OR  chiropract*  OR  dental*  OR  ( {dental hygiene} )  OR  ( {dental hygienist} )  OR  ( {dental hygienists} )  OR  ( {dental technician} )  OR  ( {dental technicians} )  dentist*  OR  dietetics*  OR  dietitian*  OR  ( {emergency medical technician} )  OR  ( {emergency medical technicians} )  OR  ( {foot therapy} )  OR  ( {foot therapist} )  OR  ( {foot therapists} )  OR  ( {hearing care professional} )  OR  ( {hearing care professionals} )  OR  medical*  OR  midwi*  OR  nurs*  OR  nutritition*  OR  ( {occupational therapy} )  OR  ( {occupational therapist} )  OR  ( {occupational therapists} )  OR  optician*  OR  optometr*  OR  orthoped*  OR  paramedic*  OR  perfusionis*  OR  pharmacy  OR  pharmacist*  OR  ( {physical therapy} )  OR  ( {physical therapist} )  OR  ( {physical therapists} )  OR  physiotherap*  OR  psycholog*  OR  ( {public health} )  OR  radiograph*  OR  roentgenograph*  OR  ( {social educator} )  OR  ( {social educators} )  OR  ( {health professional} )  OR  ( {health professionals} ) ) )  OR  ( ABS ( ( {health occupation} )  OR  ( {health occupations} )  OR  ( {health personnel} )  OR  ( {health science} )  OR  ( {health sciences} )  OR  audiolog*  OR  bioengineer*  OR  ( {biomedical engineering} )  OR  ( {cardiopulmonary technician} )  OR  ( {cardiopulmonary technicians} )  OR  chiropract*  OR  dental*  OR  ( {dental hygiene} )  OR  ( {dental hygienist} )  OR  ( {dental hygienists} )  OR  ( {dental technician} )  OR  ( {dental technicians} )  dentist*  OR  dietetics*  OR  dietitian*  OR  ( {emergency medical technician} )  OR  ( {emergency medical technicians} )  OR  ( {foot therapy} )  OR  ( {foot therapist} )  OR  ( {foot therapists} )  OR  ( {hearing care professional} )  OR  ( {hearing care professionals} )  OR  medical*  OR  midwi*  OR  nurs*  OR  nutritition*  OR  ( {occupational therapy} )  OR  ( {occupational therapist} )  OR  ( {occupational therapists} )  OR  optician*  OR  optometr*  OR  orthoped*  OR  paramedic*  OR  perfusionis*  OR  pharmacy  OR  pharmacist*  OR  ( {physical therapy} )  OR  ( {physical therapist} )  OR  ( {physical therapists} )  OR  physiotherap*  OR  psycholog*  OR  ( {public health} )  OR  radiograph*  OR  roentgenograph*  OR  ( {social educator} )  OR  ( {social educators} )  OR  ( {health professional} )  OR  ( {health professionals} ) ) )  OR  ( TITLE ( ( ( ( {health science} )  OR  ( {health sciences} )  OR  ( {biomedical engineering} )  OR  ( {cardiopulmonary technician} )  OR  ( {cardiopulmonary technicians} )  OR  ( {dental hygiene} )  OR  ( {dental hygienist} )  OR  ( {dental hygienists} )  OR  ( {dental technician} )  OR  ( {dental technicians} )  OR  ( {emergency medical technician} )  OR  ( {emergency medical technicians} )  OR  ( {foot therapy} )  OR  ( {foot therapist} )  OR  ( {foot therapists} )  OR  ( {hearing care professional} )  OR  ( {hearing care professionals} )  OR  ( {occupational therapy} )  OR  ( {occupational therapist} )  OR  ( {occupational therapists} )  OR  ( {physical therapy} )  OR  ( {physical therapist} )  OR  ( {physical therapists} )  OR  ( {public health} )  OR  ( {social educator} )  OR  ( {social educators} )  OR  ( {health professional} )  OR  ( {health professionals} ) )  W/4  {education} ) ) )  OR  ( ABS ( ( ( ( {health science} )  OR  ( {health sciences} )  OR  ( {biomedical engineering} )  OR  ( {cardiopulmonary technician} )  OR  ( {cardiopulmonary technicians} )  OR  ( {dental hygiene} )  OR  ( {dental hygienist} )  OR  ( {dental hygienists} )  OR  ( {dental technician} )  OR  ( {dental technicians} )  OR  ( {emergency medical technician} )  OR  ( {emergency medical technicians} )  OR  ( {foot therapy} )  OR  ( {foot therapist} )  OR  ( {foot therapists} )  OR  ( {hearing care professional} )  OR  ( {hearing care professionals} )  OR  ( {occupational therapy} )  OR  ( {occupational therapist} )  OR  ( {occupational therapists} )  OR  ( {physical therapy} )  OR  ( {physical therapist} )  OR  ( {physical therapists} )  OR  ( {public health} )  OR  ( {social educator} )  OR  ( {social educators} )  OR  ( {health professional} )  OR  ( {health professionals} ) )  W/4  {education} ) ) )  OR  ( TITLE ( ( ( ( {health science} )  OR  ( {health sciences} )  OR  ( {biomedical engineering} )  OR  ( {cardiopulmonary technician} )  OR  ( {cardiopulmonary technicians} )  OR  ( {dental hygiene} )  OR  ( {dental hygienist} )  OR  ( {dental hygienists} )  OR  ( {dental technician} )  OR  ( {dental technicians} )  OR  ( {emergency medical technician} )  OR  ( {emergency medical technicians} )  OR  ( {foot therapy} )  OR  ( {foot therapist} )  OR  ( {foot therapists} )  OR  ( {hearing care professional} )  OR  ( {hearing care professionals} )  OR  ( {occupational therapy} )  OR  ( {occupational therapist} )  OR  ( {occupational therapists} )  OR  ( {physical therapy} )  OR  ( {physical therapist} )  OR  ( {physical therapists} )  OR  ( {public health} )  OR  ( {social educator} )  OR  ( {social educators} )  OR  ( {health professional} )  OR  ( {health professionals} ) )  W/4  ( {student}  OR  {students} ) ) ) )  OR  ( ABS ( ( ( ( {health science} )  OR  ( {health sciences} )  OR  ( {biomedical engineering} )  OR  ( {cardiopulmonary technician} )  OR  ( {cardiopulmonary technicians} )  OR  ( {dental hygiene} )  OR  ( {dental hygienist} )  OR  ( {dental hygienists} )  OR  ( {dental technician} )  OR  ( {dental technicians} )  OR  ( {emergency medical technician} )  OR  ( {emergency medical technicians} )  OR  ( {foot therapy} )  OR  ( {foot therapist} )  OR  ( {foot therapists} )  OR  ( {hearing care professional} )  OR  ( {hearing care professionals} )  OR  ( {occupational therapy} )  OR  ( {occupational therapist} )  OR  ( {occupational therapists} )  OR  ( {physical therapy} )  OR  ( {physical therapist} )  OR  ( {physical therapists} )  OR  ( {public health} )  OR  ( {social educator} )  OR  ( {social educators} )  OR  ( {health professional} )  OR  ( {health professionals} ) )  W/4  ( {student}  OR  {students} ) ) ) )  OR  ( TITLE ( ( ( audiolog*  OR  bioengineer*  OR  chiropract*  OR  dental*  OR  dentist*  OR  dietetics*  OR  dietitian*  OR  medical*  OR  midwi*  OR  nurs*  OR  nutritition*  OR  optician*  OR  optometr*  OR  orthoped*  OR  paramedic*  OR  perfusionis*  OR  pharmacy  OR  pharmacist*  OR  physiotherap*  OR  psycholog*  OR  radiograph*  OR  roentgenograph* )  W/4  student* )  OR  ( ( audiolog*  OR  bioengineer*  OR  chiropract*  OR  dental*  OR  dentist*  OR  dietetics*  OR  dietitian*  OR  medical*  OR  midwi*  OR  nurs*  OR  nutritition*  OR  optician*  OR  optometr*  OR  orthoped*  OR  paramedic*  OR  perfusionis*  OR  pharmacy  OR  pharmacist*  OR  physiotherap*  OR  psycholog*  OR  radiograph*  OR  roentgenograph* )  W/4  educat* ) ) )  OR  ( ABS ( ( ( audiolog*  OR  bioengineer*  OR  chiropract*  OR  dental*  OR  dentist*  OR  dietetics*  OR  dietitian*  OR  medical*  OR  midwi*  OR  nurs*  OR  nutritition*  OR  optician*  OR  optometr*  OR  orthoped*  OR  paramedic*  OR  perfusionis*  OR  pharmacy  OR  pharmacist*  OR  physiotherap*  OR  psycholog*  OR  radiograph*  OR  roentgenograph* )  W/4  student* )  OR  ( ( audiolog*  OR  bioengineer*  OR  chiropract*  OR  dental*  OR  dentist*  OR  dietetics*  OR  dietitian*  OR  medical*  OR  midwi*  OR  nurs*  OR  nutritition*  OR  optician*  OR  optometr*  OR  orthoped*  OR  paramedic*  OR  perfusionis*  OR  pharmacy  OR  pharmacist*  OR  physiotherap*  OR  psycholog*  OR  radiograph*  OR  roentgenograph* )  W/4  educat* ) ) ) )  AND  ( ( ( TITLE ( ( (77) )  OR  ( {health student} )  OR  ( {health students} )  OR  graduate*  OR  undergraduate*  OR  postgraduate*  OR  bachelor*  OR  master*  OR  ( {tertiary education} )  OR  ( {postsecondary education} )  OR  college  OR  university  OR  ( {distance education} ) )  OR  ABS ( ( (77) )  OR  ( {health student} )  OR  ( {health students} )  OR  graduate*  OR  undergraduate*  OR  postgraduate*  OR  bachelor*  OR  master*  OR  ( {tertiary education} )  OR  ( {postsecondary education} )  OR  college  OR  university  OR  ( {distance education} ) ) ) )  OR  ( TITLE ( ( ( ( {health science} )  OR  ( {health sciences} )  OR  ( {biomedical engineering} )  OR  ( {cardiopulmonary technician} )  OR  ( {cardiopulmonary technicians} )  OR  ( {dental hygiene} )  OR  ( {dental hygienist} )  OR  ( {dental hygienists} )  OR  ( {dental technician} )  OR  ( {dental technicians} )  OR  ( {emergency medical technician} )  OR  ( {emergency medical technicians} )  OR  ( {foot therapy} )  OR  ( {foot therapist} )  OR  ( {foot therapists} )  OR  ( {hearing care professional} )  OR  ( {hearing care professionals} )  OR  ( {occupational therapy} )  OR  ( {occupational therapist} )  OR  ( {occupational therapists} )  OR  ( {physical therapy} )  OR  ( {physical therapist} )  OR  ( {physical therapists} )  OR  ( {public health} )  OR  ( {social educator} )  OR  ( {social educators} )  OR  ( {health professional} )  OR  ( {health professionals} ) )  W/4  {education} ) ) )  OR  ( ABS ( ( ( ( {health science} )  OR  ( {health sciences} )  OR  ( {biomedical engineering} )  OR  ( {cardiopulmonary technician} )  OR  ( {cardiopulmonary technicians} )  OR  ( {dental hygiene} )  OR  ( {dental hygienist} )  OR  ( {dental hygienists} )  OR  ( {dental technician} )  OR  ( {dental technicians} )  OR  ( {emergency medical technician} )  OR  ( {emergency medical technicians} )  OR  ( {foot therapy} )  OR  ( {foot therapist} )  OR  ( {foot therapists} )  OR  ( {hearing care professional} )  OR  ( {hearing care professionals} )  OR  ( {occupational therapy} )  OR  ( {occupational therapist} )  OR  ( {occupational therapists} )  OR  ( {physical therapy} )  OR  ( {physical therapist} )  OR  ( {physical therapists} )  OR  ( {public health} )  OR  ( {social educator} )  OR  ( {social educators} )  OR  ( {health professional} )  OR  ( {health professionals} ) )  W/4  {education} ) ) )  OR  ( TITLE ( ( ( ( {health science} )  OR  ( {health sciences} )  OR  ( {biomedical engineering} )  OR  ( {cardiopulmonary technician} )  OR  ( {cardiopulmonary technicians} )  OR  ( {dental hygiene} )  OR  ( {dental hygienist} )  OR  ( {dental hygienists} )  OR  ( {dental technician} )  OR  ( {dental technicians} )  OR  ( {emergency medical technician} )  OR  ( {emergency medical technicians} )  OR  ( {foot therapy} )  OR  ( {foot therapist} )  OR  ( {foot therapists} )  OR  ( {hearing care professional} )  OR  ( {hearing care professionals} )  OR  ( {occupational therapy} )  OR  ( {occupational therapist} )  OR  ( {occupational therapists} )  OR  ( {physical therapy} )  OR  ( {physical therapist} )  OR  ( {physical therapists} )  OR  ( {public health} )  OR  ( {social educator} )  OR  ( {social educators} )  OR  ( {health professional} )  OR  ( {health professionals} ) )  W/4  ( {student}  OR  {students} ) ) ) )  OR  ( ABS ( ( ( ( {health science} )  OR  ( {health sciences} )  OR  ( {biomedical engineering} )  OR  ( {cardiopulmonary technician} )  OR  ( {cardiopulmonary technicians} )  OR  ( {dental hygiene} )  OR  ( {dental hygienist} )  OR  ( {dental hygienists} )  OR  ( {dental technician} )  OR  ( {dental technicians} )  OR  ( {emergency medical technician} )  OR  ( {emergency medical technicians} )  OR  ( {foot therapy} )  OR  ( {foot therapist} )  OR  ( {foot therapists} )  OR  ( {hearing care professional} )  OR  ( {hearing care professionals} )  OR  ( {occupational therapy} )  OR  ( {occupational therapist} )  OR  ( {occupational therapists} )  OR  ( {physical therapy} )  OR  ( {physical therapist} )  OR  ( {physical therapists} )  OR  ( {public health} )  OR  ( {social educator} )  OR  ( {social educators} )  OR  ( {health professional} )  OR  ( {health professionals} ) )  W/4  ( {student}  OR  {students} ) ) ) )  OR  ( TITLE ( ( ( audiolog*  OR  bioengineer*  OR  chiropract*  OR  dental*  OR  dentist*  OR  dietetics*  OR  dietitian*  OR  medical*  OR  midwi*  OR  nurs*  OR  nutritition*  OR  optician*  OR  optometr*  OR  orthoped*  OR  paramedic*  OR  perfusionis*  OR  pharmacy  OR  pharmacist*  OR  physiotherap*  OR  psycholog*  OR  radiograph*  OR  roentgenograph* )  W/4  student* )  OR  ( ( audiolog*  OR  bioengineer*  OR  chiropract*  OR  dental*  OR  dentist*  OR  dietetics*  OR  dietitian*  OR  medical*  OR  midwi*  OR  nurs*  OR  nutritition*  OR  optician*  OR  optometr*  OR  orthoped*  OR  paramedic*  OR  perfusionis*  OR  pharmacy  OR  pharmacist*  OR  physiotherap*  OR  psycholog*  OR  radiograph*  OR  roentgenograph* )  W/4  educat* ) ) )  OR  ( ABS ( ( ( audiolog*  OR  bioengineer*  OR  chiropract*  OR  dental*  OR  dentist*  OR  dietetics*  OR  dietitian*  OR  medical*  OR  midwi*  OR  nurs*  OR  nutritition*  OR  optician*  OR  optometr*  OR  orthoped*  OR  paramedic*  OR  perfusionis*  OR  pharmacy  OR  pharmacist*  OR  physiotherap*  OR  psycholog*  OR  radiograph*  OR  roentgenograph* )  W/4  student* )  OR  ( ( audiolog*  OR  bioengineer*  OR  chiropract*  OR  dental*  OR  dentist*  OR  dietetics*  OR  dietitian*  OR  medical*  OR  midwi*  OR  nurs*  OR  nutritition*  OR  optician*  OR  optometr*  OR  orthoped*  OR  paramedic*  OR  perfusionis*  OR  pharmacy  OR  pharmacist*  OR  physiotherap*  OR  psycholog*  OR  radiograph*  OR  roentgenograph* )  W/4  educat* ) ) ) )  AND  ( ( TITLE ( digital*  OR  online*  OR  virtual*  OR  blended  OR  hybrid  OR  distance  OR  synchron*  OR  asynchron*  OR  decentral*  OR  ( {e learning} )  OR  ( {e-learning} )  OR  ( {electronic learning} )  OR  ( {electronic-learning} )  OR  ( {computer supported} )  OR  ( {computer-supported} ) )  OR  ABS ( digital*  OR  online*  OR  virtual*  OR  blended  OR  hybrid  OR  distance  OR  synchron*  OR  asynchron*  OR  decentral*  OR  ( {e learning} )  OR  ( {e-learning} )  OR  ( {electronic learning} )  OR  ( {electronic-learning} )  OR  ( {computer supported} )  OR  ( {computer-supported} ) ) ) )  AND  ( ( TITLE ( ( {Collaborative learning} )  OR  ( {group work} )  OR  ( {small group*} )  OR  ( {peer group*} )  OR  ( {cooperative learning} )  OR  ( {team learning} )  OR  ( {tutorial group*} )  OR  ( {peer learning} )  OR  ( {group learning} )  OR  ( {student group*} )  OR  ( {group based} )  OR  ( {team based} )  OR  ( {peer assisted learning} )  OR  ( {peer assisted} ) )  OR  ABS ( ( {Collaborative learning} )  OR  ( {group work} )  OR  ( {small group*} )  OR  ( {peer group*} )  OR  ( {cooperative learning} )  OR  ( {team learning} )  OR  ( {tutorial group*} )  OR  ( {peer learning} )  OR  ( {group learning} )  OR  ( {student group*} )  OR  ( {group based} )  OR  ( {team based} )  OR  ( {peer assisted learning} )  OR  ( {peer assisted} ) ) ) ) |  |

Epistemonikos

(title:(digital* OR online* OR "e-learning" OR "e learning" OR "electronic learning" OR "electronic-learning" OR "distance learning") OR abstract:(digital* OR online* OR "e-learning" OR "e learning" OR "electronic learning" OR "electronic-learning" OR "distance learning")) AND (title:("Group learning" OR "collaborative learning" OR "cooperative learning" OR "small group" OR "small groups" OR "group work") OR abstract:("Group learning" OR "collaborative learning" OR "cooperative learning" OR "small group" OR "small groups" OR "group work")) AND (title:(education OR student*) OR abstract:(education OR student*)) AND (title:("health occupation" OR "health personnel" OR "health science" OR chiropract* OR dietetic* OR dietitian* OR dentist* OR dental OR midwi* OR nurs* OR medical* OR "occupational therapy" OR "occupational therapist" OR paramedic* OR pharmacy OR pharmacist* OR psycholog* OR "physical therapy" OR "physical therapist" OR "public health" OR "health professional" OR "health professionals") OR abstract:("health occupation" OR "health personnel" OR "health science" OR chiropract* OR dietetic* OR dietitian* OR dentist* OR dental OR midwi* OR nurs* OR medical* OR "occupational therapy" OR "occupational therapist" OR paramedic* OR pharmacy OR pharmacist* OR psycholog* OR "physical therapy" OR "physical therapist" OR "public health" OR "health professional" OR "health professionals"))

Limiters: Limiters - Published Date: 20120901-20220831; Language: Danish, English, Norwegian, Swedish

# Appendix 2 Data charting form

| **Reference/ article characteristics** | |
| --- | --- |
| First author |  |
| Title |  |
| Journal reference |  |
| **Description of Study** | |
| Purpose/aim of study |  |
| Design |  |
| Method for inclusion of participants |  |
| Description of the methods for data analysis |  |
| **Description of study population** | |
| Higher education level/degree |  |
| Study program/subject - Health Science |  |
| Country |  |
| Number of participants |  |
| Age |  |
| Gender |  |
| **Description of concept** | |
| Type of assignment (e.g., discussion, case, exam, written, oral, presentation, essay) |  |
| Format of delivery |  |
| Duration of group work (e.g., hours, sessions, weeks) |  |
| Group size |  |
| Part of a learning model if applicable (e.g., TBL, PBL, CBL, IPL etc.) |  |
| Description/definition of group work (e.g., group work, collaboration, |  |
| Description of intervention/action related to digital group work |  |
| **Description of context** | |
| Digital format (e.g., synchronous, asynchronous, blended, hybrid) |  |
| Digital communication and collaboration tools |  |
| Digital learning tools/collaboration |  |
| Description/definition of digital (e.g., e-learning, online learning, distance education, remote education, virtual learning) |  |
| **Outcomes (results or findings)** | |
| Main outcome |  |
| Outcome related to digital group work (e.g., effect, satisfaction, experience, perception, motivation) |  |
| **Facilitators or barriers to digital group work in relation to CoI by Garrison (2000, 2016)** | |
| Teaching presence |  |
| Social presence |  |
| Cognitive presence |  |
| **Facilitators or barriers to digital group work outside CoI** | |
|  |  |
|  |  |
|  |  |
| **Teacher/faculty also the researcher (dual role)?** | |
| **Yes/No/Not stated** |  |
| If Yes/No: Stated in the article; discussed in the article; stated in author’s contributions |  |
| **Inclusion recommended Yes/No** | |
|  | |

# Appendix 3 List of consulted stakeholders

| **Name** | **Title** | **Affiliation** |
| --- | --- | --- |
| Hege Hermansen | Associate Professor | Oslo Metropolitan University, [Centre for the Study of Professions](https://www.oslomet.no/en/about/employee-directory?unitcode=sps&departmentcode=sps) –[Teaching and Research](https://www.oslomet.no/en/about/employee-directory?unitcode=sps&departmentcode=sps-u) |
| Karoline Jøranli Thomlevold | Specialized Health Care Services Physiotherapist | Falck Norway |
